# The virome of the panglobal, wide host-range plant pathogen *Phytophthora cinnamomi*: phylogeography and evolutionary insights

**DOI:** 10.1093/ve/veaf020

**Published:** 2025-04-01

**Authors:** Leticia Botella, Ondřej Hejna, Tomáš Kudláček, Kateřina Kovačiková, Michael Rost, Marco Forgia, Milica Raco, Ivan Milenković, Tamara Corcobado, Cristiana Maia, Bruno Scanu, André Drenth, David I Guest, Edward C Y Liew, Nguyen Minh Chi, Pham Quang Thu, Tun-Tschu Chang, Chuen-Hsu Fu, Koji Kageyama, Ayaka Hieno, Hayato Masuja, Seiji Uematsu, Álvaro Durán, Marthin Tarigan, Muhammad Junaid, Nasri Nasri, Eugenio Sanfuentes, Vladislav Čurn, Joan F Webber, Clive M Brasier, Marília Horta Jung, Thomas Jung

**Affiliations:** Department of Forest Protection and Wildlife Management, Faculty of Forestry and Wood Technology, Mendel University in Brno, Zemědělská 3, Brno 613 00, Czech Republic; Phytophthora Research Centre, Department of Forest Protection and Wildlife Management, Faculty of Forestry and Wood Technology, Mendel University in Brno, Zemědělská 3, Brno 613 00, Czech Republic; Department of Genetics and Agrobiotechnology, Faculty of Agriculture and Technology, University of South Bohemia in České Budějovice, Na Sádkách 1780, České Budějovice 370 05, Czech Republic; Phytophthora Research Centre, Department of Forest Protection and Wildlife Management, Faculty of Forestry and Wood Technology, Mendel University in Brno, Zemědělská 3, Brno 613 00, Czech Republic; Institute for Mathematics and Computer Science & Center for Functional Genomics of Microbes, University of Greifswald, Walther-Rathenau-Straße 47, Greifswald 17489, Germany; Department of Forest Protection and Wildlife Management, Faculty of Forestry and Wood Technology, Mendel University in Brno, Zemědělská 3, Brno 613 00, Czech Republic; Department of Genetics and Agrobiotechnology, Faculty of Agriculture and Technology, University of South Bohemia in České Budějovice, Na Sádkách 1780, České Budějovice 370 05, Czech Republic; Institute for Sustainable Plant Protection, National Research Council of Italy, Strada delle Cacce 73 - 10135, Torino 10135, Italy; Phytophthora Research Centre, Department of Forest Protection and Wildlife Management, Faculty of Forestry and Wood Technology, Mendel University in Brno, Zemědělská 3, Brno 613 00, Czech Republic; Department of Genetics and Agrobiotechnology, Faculty of Agriculture and Technology, University of South Bohemia in České Budějovice, Na Sádkách 1780, České Budějovice 370 05, Czech Republic; Department of Genetics and Agrobiotechnology, Faculty of Agriculture and Technology, University of South Bohemia in České Budějovice, Na Sádkách 1780, České Budějovice 370 05, Czech Republic; Centre of Marine Sciences (CCMAR), University of Algarve, Gambelas Campus 8005-139, Faro 8005-139, Portugal; Department of Agricultural Sciences, University of Sassari, Viale Italia 39A, Sassari 07100, Italy; Centre for Horticultural Science, The University of Queensland, Ecosciences Precinct, 41 Boggo Road, Dutton Park, Brisbane, Qld 4001, Australia; Sydney Institute of Agriculture, School of Life and Environmental Sciences, The University of Sydney, NSW 2006, Australia; Research Centre for Ecosystem Resilience, Australian Institute of Botanical Science, The Royal Botanic Gardens and Domain Trust, Mrs Macquaries Rd, Sydney, NSW 2006, Australia; Forest Protection Research Centre, Vietnamese Academy of Forest Sciences, 46 Duc Thang Road, Hanoi 10000, Vietnam; Forest Protection Research Centre, Vietnamese Academy of Forest Sciences, 46 Duc Thang Road, Hanoi 10000, Vietnam; Forest Protection Division, Taiwan Forestry Research Institute, No. 53, Nanhai Rd, Taipei, Taiwan; Forest Protection Division, Taiwan Forestry Research Institute, No. 53, Nanhai Rd, Taipei, Taiwan; Center for Environmental and Societal Sustainability, Gifu University, Gifu 501-1193, Japan; Center for Environmental and Societal Sustainability, Gifu University, Gifu 501-1193, Japan; Forestry and Forest Products Research Institute (FFPRI), Ibaraki, Tsukuba, IB 305-8687, Japan; Department of Bioregulation and Biointeraction, Laboratory of Molecular and Cellular Biology, Tokyo University of Agriculture and Technology, Fuchu, TK 183-8509, Japan; Fiber Research and Development, Asia Pacific Resources International Limited (APRIL), Kabupaten Pelalawan, Pangkalan Kerinci, RI 28300, Indonesia; Fiber Research and Development, Asia Pacific Resources International Limited (APRIL), Kabupaten Pelalawan, Pangkalan Kerinci, RI 28300, Indonesia; Department of Plant Pest and Disease, Faculty of Agriculture, Hasanuddin University, Jalan Perintis Kemerdekaan Km. 10, Makassar, SN 90245, Indonesia; Faculty of Forestry, Hasanuddin University, Jalan Perintis Kemerdekaan km.10, Makassar, SN 90245, Indonesia; Laboratorio de Patología Forestal, Facultad Ciencias Forestales y Centro de Biotecnología, Universidad de Concepción, Barrio Universitario s/n, Concepción 4030000, Chile; Department of Genetics and Agrobiotechnology, Faculty of Agriculture and Technology, University of South Bohemia in České Budějovice, Na Sádkách 1780, České Budějovice 370 05, Czech Republic; Forest Research, Alice Holt Lodge, GU10 4LH, Farnham, Surrey, United Kingdom; Forest Research, Alice Holt Lodge, GU10 4LH, Farnham, Surrey, United Kingdom; Phytophthora Research Centre, Department of Forest Protection and Wildlife Management, Faculty of Forestry and Wood Technology, Mendel University in Brno, Zemědělská 3, Brno 613 00, Czech Republic; Phytophthora Research Centre, Department of Forest Protection and Wildlife Management, Faculty of Forestry and Wood Technology, Mendel University in Brno, Zemědělská 3, Brno 613 00, Czech Republic

**Keywords:** forest emerging diseases, oomycetes, virus diversity, virus evolution, virus–host coevolution, HTS orphan contigs, mating

## Abstract

*Phytophthora cinnamomi* stands out as one of the most devastating plant pathogens worldwide, rapidly expanding its range and impacting a wide range of host species. In this study, we investigated the virome of *P. cinnamomi* across 222 isolates from Africa, Asia, Europe, Oceania, and the Americas using stranded total RNA sequencing, reverse transcription polymerase chain reaction screening, and Sanger sequencing of selected isolates. Our analysis revealed that virus infections were prevalent across all sampled populations, including RNA viruses associated with the orders *Ghabrivirales, Martellivirales*, and *Tolivirales*, and the classes *Amabiliviricetes, Bunyaviricetes*, and the recently proposed *Orpoviricetes*. Viruses were mainly found in East and Southeast Asian populations, within the geographic origin of *P. cinnamomi* but have also spread to new regions where the pathogen has emerged as a clonal destructive pathogen. Among the identified viruses, eight species, including two bunya-like viruses, one narna-like virus, and five ormycoviruses, exhibit a global distribution with some genetic divergence between continents. The interaction between *P. cinnamomi* and its virome indicates a dynamic coevolution across diverse geographic regions. Indonesia is indicated to be the viral epicentre of *P. cinnamomi*, with the highest intra- and interspecies diversity of viruses. Viral diversity is significantly enhanced in regions where sexual recombination of *P. cinnamomi* occurs, while regions with predominantly asexual reproduction harbour fewer viral species. Interestingly, only the partially self-fertile mating type (MAT) A2, associated with the global pandemic, facilitates the spread of viruses across different biogeographic regions, whereas viruses are absent in the self-sterile MAT A1 in its areas of introduction like Australia and South Africa. Intriguingly, the presence of a plant tombusvirus suggests a potential cross-kingdom infection among Chilean isolates and a plant host. This study sheds further light on the geographical origin of *P. cinnamomi* from a novel virome perspective.

## Introduction

*Phytophthora cinnamomi* Rands is a panglobal and invasive soil-borne oomycete pathogen phylogenetically classified within Clade 7c of the genus *Phytophthora* belonging to the kingdom Straminipila (Beakes et al. 2014; Erwin and Ribeiro, 1998; Chen et al. 2022). Originating from Southeast Asia (Ko et al. 1978; Shakya et al. 2021), where it usually does not cause severe diseases of natural vegetation (Jung et al. 2017, 2020), it has emerged as one of the most destructive plant pathogens globally. It has the potential to induce root rot, dieback, cankers, and mortality in over 5000 woody species, including numerous vital crops, ornamental plants, and trees (Kamoun et al. 2015; Hardham and Blackman 2018). Some of the most notable diseases occur in North American and European chestnut, pine and oak forests, *Araucaria* forests in Brazil and Chile, and Valdivian rainforests (Oak and Tainter 1988; Brasier et al. 1993; Jung and Dobler 2002; Dos Santos et al. 2011; McConnell and Balci 2014; Jung et al. 2018a, 2018b; Sanfuentes et al. 2022). It also severely affects avocado and macadamia crops worldwide (Reeksting et al. 2014; Akinsanmi et al. 2017) and natural vegetation, including eucalypt forests and Banksia woodlands in southeastern and southwestern Australia (Erwin and Ribeiro, 1998; Weste 2003; Jung et al. 2013; Beaulieu et al. 2017; Burgess et al. 2017; Jung et al. 2018b).

As other plant pathogen invasions the rapid spread and introduction of *P. cinnamomi* to new territories throughout the world has been facilitated by global trade and the unintentional movement of contaminated nursery stock and other plant material (Santini et al. 2013; Jung et al. 2016; Horta Jung et al. 2024). *Phytophthora cinnamomi* is a heterothallic species requiring the presence of two mating types, A1 and A2, for sexual reproduction, which occurs through the formation of oospores (Erwin and Ribeiro 1998). Although both mating types have been found around the world, the global epidemic is driven by two clonal lineages, PcG1-A2 and PcG2-A2, of mating type (MAT) A2 (Linde et al. 1999; Dobrowolski et al. 2003; Eggers et al. 2012; Beaulieu et al. 2017; Engelbrecht et al. 2017; Serrano et al. 2019; Shakya et al. 2021). Only in its geographic origin, mainly in Taiwan and Vietnam, *P. cinnamomi* populations have an equal ratio of A1 and A2 mating types, increasing the likelihood of encounters between compatible types (Pratt et al. 1973; Shepherd 1975; Arentz and Simpson 1986; Ko et al. 1978; Arentz 2017; Jung et al. 2017, 2020; Shakya et al. 2021).

In recent times, an escalating number of ‘ooviruses’ or ‘oomycoviruses’ (Forgia et al. 2024) have been documented within the *Phytophthora* genus. Both singular and multiple virus infections appear to be prevalent. Among these, double-stranded (ds) RNA viruses associated with the families *Orthototiviridae, Ootiviridae, Pseudototiviridae, Megabirnaviridae, Botybirnaviridae, Fusagraviridae*, positive (+) single-stranded (ss) RNA viruses linked to the *Tombusviridae, Narnaviridae*, and *Endornaviridae*, and negative (−) ssRNA viruses related to the class *Bunyaviricetes* have been identified in various *Phytophthora* species, including *P. cactorum, P. castaneae, P. condilina, P. heveae, P. infestans, P. pluvialis*, the ‘*P. palustris*’ complex, *P. ramorum*, and an unidentified species infecting *Asparagus officinalis* in Japan (Hacker et al. 2005; Cai and Hillman 2013; Botella and Jung 2021; Poimala et al. 2021; Uchida et al. 2021; Botella et al. 2022; Raco et al. 2022, 2023; Xu et al. 2022). However, the consequences of these virus–host relationships remain ambiguous. *Phytophthora* sp. isolates from Japan, co-infected with two endornaviruses, exhibited a reduction in growth rate and hyphal density, accompanied by an increased production of zoosporangia and alterations in sensitivity to fungicides (Uchida et al. 2021). Likewise, Phytophthora infestans RNA virus 2 (PiRV-2) has been identified to enhance zoosporangium formation in *P. infestans* (Cai et al. 2019). Furthermore, co-infection with Phytophthora cactorum bunyavirus 1 and 2 reduces *P. cactorum* growth in apple fruit tissues and *in vitro*, decreases sporangia production and size, and increases elicitin production, but does not alter *P. cactorum* pathogenicity in planta (Poimala et al. 2021).

Over the course of millions of years, viruses have undergone coevolution alongside their natural hosts. Both entities coexist as integral components of a holobiont within ecosystems, emphasizing their interconnectedness rather than isolated existence (Lefeuvre et al. 2019). The impact of viruses on host behaviour and, consequently, on host populations is likely a result of intricate interactions within the complex environment–host–virus relationships (Poisot et al. 2015). In native environments, it is anticipated that antagonists, including hyperparasites such as viruses, would be more prevalent compared to other settings (Roderick and Navajas 2003; Prospero et al. 2021; Shamsi et al. 2024). These antagonists are expected to play a crucial role in disease control. However, the impact of viruses on host behaviour (i.e. virulence of pathogens) in newly introduced areas, especially in the context of emerging forest diseases, remains unpredictable. In the case of fungal and oomycete viruses, replication and transmission occur intracellularly, horizontally between individuals or vertically from parent to offspring, hence, potential parallel evolution with their hosts is hinted (Goker et al. 2011). Consequently, host diversification, resulting from geographical separation and ecological adaptation, is reflected in viral communities (Bryner et al. 2012; Botella et al. 2015, 2022; Schoebel et al. 2017).

In this context and following up on the question raised by Hardham and Blackman (2018) ‘What aspects of *P. cinnamomi* molecular or cellular make-up would be good targets for novel, specific and sustainable control measures?’, we decided to (i) study and describe the virome of *P. cinnamomi* in a global and representative collection of isolates; and (ii) compare the virus diversity and abundance in native and non-native habitats of *P. cinnamomi*; in order to (iii) narrow-down its specific centre of origin.

## Materials and methods

### Sampling and isolation of *P. cinnamomi*

A total of 222 *P. cinnamomi* isolates including 10 from Africa (Algeria, South Africa, and Tunisia), 129 from Asia (Japan, Indonesia, Taiwan, and Vietnam), 36 from Oceania (Australia and Papua New Guinea), 32 from Europe (Austria, France, Italy, Hungary, Portugal, and Spain), 7 from North America (Dominican Republic and USA), and 8 from South America (Chile) were collected from rhizosphere soil samples using various baiting approaches and by plating of infected root or bark tissue onto *Phytophthora*-selective agar media (Jung et al. 2016). Except for 51 isolates mainly collected from Japan and Indonesia, 171 of the isolates included in this study were previously used in the population genomic study of *P. cinnamomi* by Shakya et al. (2021). In total, 141 MAT A2 isolates, 77 MAT A1 isolates, 3 sterile isolates, and 1 isolate with unknown MAT were studied. A comprehensive list of *P. cinnamomi* isolates used in this study and details of their sampling locations, host plants, dates, and collectors are given in Supplementary Table S1.

### RNA extraction

Total RNA was purified from ∼100 mg of 7–10-day-old mycelium using RNAzol® RT Column Kit (Chomczynski et al. 2010) and treated with TURBO DNA-free™ Kit (Ambion). RNA was quantified in Qubit® 2.0 Fluorometer (Invitrogen), and quality was tested by Tape Station 4200 (Agilent). A total of 21 pools were prepared according to the host’s continental, country and region of origin, and the number of available isolates (Tables 1 and S1). The smallest (P04) and the largest (P09) pools contain 4 and 17 RNA, respectively. P03 contains RNA from isolates from two continents, Europe (Spain) and Africa (Algeria and Tunisia), but all of them from Mediterranean region. Similarly, P04 contains RNA from Oceania (Papua New Guinea) and Asia (Indonesia). Our criterion was to pool up to a maximum of 20 RNA samples to balance sequencing depth and sensitivity, minimize complexity, and align with standard practices from our previous virome studies (Botella et al. 2022). This limit ensures adequate representation of low-abundance viruses while maintaining technical consistency across samples. Only the two USA isolates were not subjected to RNA sequencing, but they were later added to the virus screening study.

**Table 1.** Detailed information for representative viruses characterized in this study.

Genome	Acronym^a^	Final contig/variant name	Genomic segment	Accession number	Contig length (nt)	Reads^d^	Mean depth^e^	Most similar virus in GenBank (BLASTX)	E-value	Identity (%)	QC (%)	CDD-search^f^
**(+)ssRNA**	PciAV1	variant 1_Vietnamese	RNA1	PP891628	2880	296	14	Setosphaeria turcica ambiguivirus 2	0.0	52.01	72	ps_ssRNAv_Tolivirales_RdRp RdRP_3
	PciTbLV1	variant 1_Chilean	RNA1	PP891944	4111	3333	108	Bermuda grass latent virus	0.0	99	68	Panicovirus_RdRp RdRP_3 Tombus_movement super family Viral_coat (S domain)
	PciAEV1	variant 1_Sumatran	RNA1	PP891625	12 807	41 284	415	Phytophthora cactorum alphaendornavirus 2	0.0	58.13	98	Endornaviridae_RdRp RdRP_2 Viral_helicase1
	PciAEV2	variant 1_SLJV^c^	RNA1	PP891627	12 843	13 775	137	Phytophthora heveae alphaendornavirus 1	0.0	42.82	98	Endornaviridae_RdRp RdRP_2 Viral_helicase1
	PciNLV1	variant_1_Portuguese	RNA1	PP891690	2415	870 295	467	Phytophthora palustris narna-like virus 5	5.00E-46	31.36	53	ps-ssRNAv_Narnaviridae_RdRp
	PciNLV2	variant 8_European	RNA1	PP891698	3128	142 799	5583	Phytophthora castaneae RNA virus 2	0.0	71.59	83	ps-ssRNAv_Narnaviridae_RdRp
	PciNLV3	variant 1_Taiwanese	RNA1	PP891704	3099	1620	65	Phytophthora castaneae RNA virus 2	0.0	80.39	83	ps-ssRNAv_Narnaviridae_RdRp
	PciNLV4	variant 1_Taiwanese	RNA1	PP891705	2556	20 228	911	Phytophthora castaneae RNA virus 2	0.0	72.06	97	ps-ssRNAv_Narnaviridae_RdRp
	PciNLV5	variant 1_Vietnamese	RNA1	PP891707	3065	55 948	2253	Phytophthora castaneae RNA virus 2	0.0	83.56	89	ps-ssRNAv_Narnaviridae_RdRp
	PciNLV6	variant 3_Sumatran	RNA1	PP891710	3051	179 627	7358	Phytophthora castaneae RNA virus 2	0.0	82.87	85	ps-ssRNAv_Narnaviridae_RdRp
	PciNLV7	variant 1_SLJV^c^	RNA1	PP891711	1968	30 744	1945	Phytophthora castaneae RNA virus 2	0.0	81.31	95	ps-ssRNAv_Narnaviridae_RdRp
	PciNLV8	variant 1_Sumatran	RNA1	PP891712	3627	43 841	1519	Erysiphe necator-associated narnavirus 8	0.0	42.12	88	No significant similarity found
**dsRNA**	PciCV1	variant 1_Sumatran	dsRNA1	PP891685	3465	301	12	Aspergillus fumigatus chrysovirus_RdRP	0.0	91.66	96	RdRP_4
			dsRNA2	PP891682	3039	256	12	Aspergillus fumigatus chrysovirus_CP	0.0	82.98	93	No significant similarity found
			dsRNA3	PP891683	2789	238	11	Aspergillus fumigatus chrysovirus_P3	0.0	79.41	94	No significant similarity found
			dsRNA4	PP891684	2573	232	9	Aspergillus fumigatus chrysovirus_P4	0.0	92.68	93	No significant similarity found
	PciTLV1	variant 1_Vietnamese	dsRNA1	PP891945	5454	1119	26	Phytophthora cactorum RNA virus 1	0.0	77.48	81	RdRP_4
	PciTLV2	variant 1_Sulawesi	dsRNA1	PP891946	6294	2210	45	Trichoderma harzianum dsRNA virus 3	0.0	43.29	43	RdRP_4
	PciFLV1	variant 1_Vietnamese	dsRNA1	PP891686	7003	36 671	661	Utsjoki toti-like virus	1E-40	28.75	25	RdRP_4
	PciFLV2	variant 1_Sumatran	dsRNA1	PP891688	6942	32 029	570	Phytophthora castaneae RNA virus 4	0.0	69.69	52	RdRP_4
**(-)ssRNA**	PciBLV1	variant 1_Vietnamese	RNA1	PP891629	9086	17 085	245	Phytophthora condilina negative-stranded RNA virus 11	0.0	65.84	98	Bunya_RdRp
	PciBLV2	variant 1_Taiwanese	RNA1	PP891633	9088	23 273	326	Phytophthora condilina negative*-*stranded RNA virus 11	0.0	65.35	98	Bunya_RdRp
	PciBLV3	variant 8_European	RNA1	PP891643	9194	96 735	1073	Phytophthora condilina negative-stranded RNA virus 11	0.0	65.67	96	Bunya_RdRp
	PciBLV4	variant 6_Australian	RNA1	PP891656	9084	29 342	431	Phytophthora condilina negative-stranded RNA virus 11	0.0	65.37	98	Bunya_RdRp
	PciBLV5	variant 1_Papuan	RNA1	PP891662	9109	50 454	724	Phytophthora condilina negative-stranded RNA virus 11	0.0	64.56	96	Bunya_RdRp
	PciBLV6	variant 1_Vietnamese	RNA1	PP891663	9417	11 228	156	Phytophthora palustris bunya-like virus 12	0.0	59.47	99	Bunya_RdRp
	PciBLV7	variant 1_Taiwanese	RNA1	PP891664	9414	13 532	188	Phytophthora palustris bunya-like virus 12	0.0	58.84	99	Bunya_RdRp
	PciBLV8	variant 2_Taiwanese	RNA1	PP891666	8989	27 076	387	Sanya bunya-like virus 10	0.0	47.84	86	Bunya_RdRp
	PciBLV9	variant 1_Vietnamese	RNA1	PP891667	8354	15 127	236	Phytophthora palustris bunya-like virus 5	0.0	72.75	99	Bunya_RdRp
	PciBLV10	variant 2_Taiwanese	RNA1	PP891669	8396	27 027	424	Phytophthora palustris bunya-like virus5	0.0	73.80	98	Bunya_RdRp
	PciBLV11	variant 1_Taiwanese	RNA1	PP891670	8408	17 669	264	Phytophthora palustris bunya-like virus 5	0.0	72.54	98	Bunya_RdRp L protein_N terminus
	PciBLV12	variant 1_Taiwanese	RNA1	PP891671	8353	2612	39	Phytophthora palustris bunya-like virus 12	0.0	58.84	99	Bunya_RdRp
	PciBLV13	variant 1_Taiwanese	RNA1	PP891672	8357	35 235	548	Phytophthora palustris bunya-like virus 5	0.0	72.92	99	Bunya_RdRp
	PciBLV14	variant 1_Sumatran	RNA1	PP891673	8359	8753	136	Phytophthora palustris bunya-like virus 5	0.0	73.52	99	Bunya_RdRp
	PciBLV15	variant 1_SLJV^c^	RNA1	PP891674	8347	6192	96	Phytophthora palustris bunya-like virus 5	0.0	73.29	99	Bunya_RdRp L protein_N terminus
	PciBLV16	variant 2_Sumatran	RNA1	PP891676	9089	10 327	147	Phytophthora condilina negative-stranded RNA virus 11	0.0	65.41	98	Bunya_RdRp
	PciBLV17	variant 1_Taiwanese	RNA1	PP891677	8431	9769	148	Phytophthora condilina negative-stranded RNA virus 11	0.0	66.68	98	Bunya_RdRp
	PciBLV18	variant 1_Taiwanese	RNA1	PP891652	9093	56 330	823	Phytophthora condilina negative-stranded RNA virus 11	0.0	65.37	98	Bunya_RdRp
**ssRNA^b^**	PciOMV1^h^	Chilean_pc01_2_c24	RNA1	PP891751	3226	403 037	15 903	Verticillium dahliae ormycovirus 1	1E-48	28.92	51	No significant similarity found
		Chilean_pc01_5_c28	RNA2	PP891713	1480	360 362	32 126	No significant similarity found				
	PciOMV2^h^	Australian_pc18_1_c17	RNA1	PP891801	3332	251 305	9872	Verticillium dahliae ormycovirus 1	7E-62	29.56	56	
		Australian_pc18_7_c15	RNA2	PP891774	1486	307 936	27 392	Verticillium dahliae ormycovirus 1	3E-18	28.65	72	
	PciOMV3^h^	Vietnamese_pc07_5_c21	RNA1	PP891825	3086	36 269	3393	Verticillium dahliae ormycovirus 1	4E-51	26.10	75	
		Vietnamese_pc07_c31	RNA2	PP891808	1418	26 495	1115	Verticillium dahliae ormycovirus 1	0.032	23.87	60	
	PciOMV4	Vietnamese_pc07_6_c11	RNA1	PP891842	3052	14 242	610	Plasmopara viticola lesion-associated ormycovirus 1	4E-16	25.05	47	
		Vietnamese_pc07_8_c50	RNA2	PP891839	1904	131 368	9042	No significant similarity found				
	PciOMV5	Kalimantan_pc14_7_c12	RNA1	PP891849	3094	10 156	429	Plasmopara viticola lesion-associated ormycovirus 1	9E-13	24.76	39	
		Kalimantan_pc14_13_c51	RNA2	PP891846	1900	27 781	1894	No significant similarity found				
	PciOMV6^h^	Sulawesian_pc14_10_c19	RNA1	PP891858	2858	7352	335	No significant similarity found				
		Sulawesian_pc14_15_c32	RNA2	PP891851	1790	3930	281	No significant similarity found				
	PciOMV7	European_pc06_4_c18	RNA1	PP891879	3206	112 141	4480	Plasmopara viticola lesion-associated ormycovirus 2	4E-15	27.93	36	
		European_pc06_7_c30	RNA2	PP891862	1791	150 438	10 766	No significant similarity found				
	PciOMV8	Sumatran_pc15_4_c35	RNA1	PP891894	3113	5990	246	Erysiphe lesion-associated ormycovirus 1	3E-14	28.66	29	
	PciOMV9^h^	Japanese_pc21_2_c29	RNA1	PP891926	3171	22 114	909	Erysiphe lesion-associated ormycovirus 1	1E-74	35.52	47	
		Japanese_pc21_6_c3	RNA2	PP891910	1518	15 328	1322	Erysiphe lesion-associated ormycovirus 1	2E-11	23.51	55	
	PciOMV10	Sumatran_pc13_5_c26	RNA1	PP891929	3166	6110	249	Erysiphe lesion-associated ormycovirus 1	7E-71	33.58	49	
	PciOMV11	Taiwanese_pc10_2_c20	RNA1	PP891940	2860	5058	225	Verticillium dahliae ormycovirus 1	0.000004	23.68	30	
		Taiwanese_pc10_9_c22	RNA2	PP891934	1889	6347	419	No significant similarity found				
	PciMOMV1^g^	Sumatran_pc15_1_c19	RNA1	PP891689	4638	85 039	2255	No significant similarity found				

Note: Due to the extensive number of viral variants/contigs identified, complete data are provided in Table S4.

**Figure 1.**
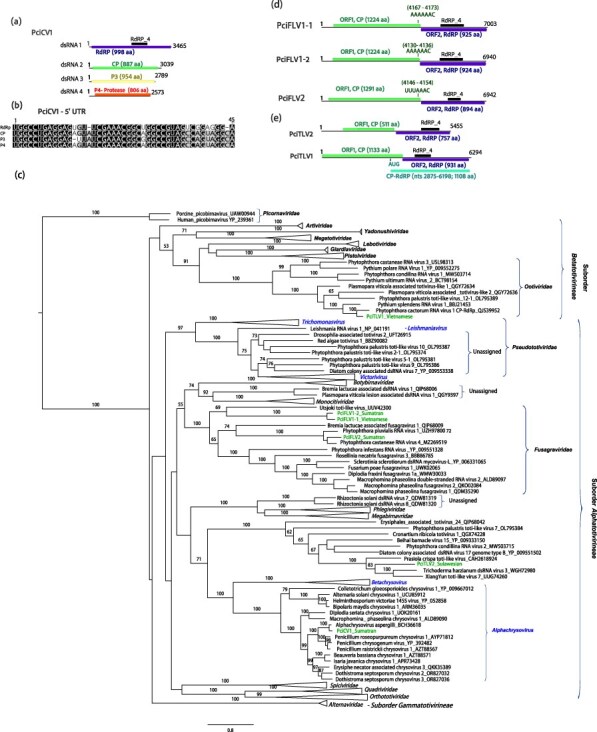
**Description of *P. cinnamomi* dsRNA viruses**. (**a**) Representation of the genome of Phytophthora cinnamomi chrysovirus 1 (PciCV1). (**b**) Sequence alignment of conserved 5ʹ-terminal regions of the four PciCV1 dsRNA segments. (**c**) Graphical representation of the genome of Phytophthora cinnamomi fusagra-like viruses (PciFLV1-3) and toti-like viruses (PciTLV1-2). (**d**) Black boxes represent the conserved domains detected, colourful boxes represent the ORFs. (**e**) Randomized Axelerated ML tree (RAxML) depicting the phylogenetic relationships of the predicted RdRP of Phytophthora cinnamomi toti-like viruses, fusagra-like viruses, and chrysovirus with other complete RdRP belonging to related viruses. *P. cinnamomi*’s viruses are abbreviated. The geographical origin of the *P. cinnamomi* isolates, where each virus and its variants were detected, is intentionally incorporated into their names. Nodes are labelled with bootstrap support values ≥50%. Branch lengths are scaled to the expected underlying number of aa substitutions per site. Some of the classified families and genera appear to simplify the tree. Family classification and the corresponding GenBank accession numbers are shown next to the virus names. Scale bars represent expected changes per site per branch.

**Figure 2.**
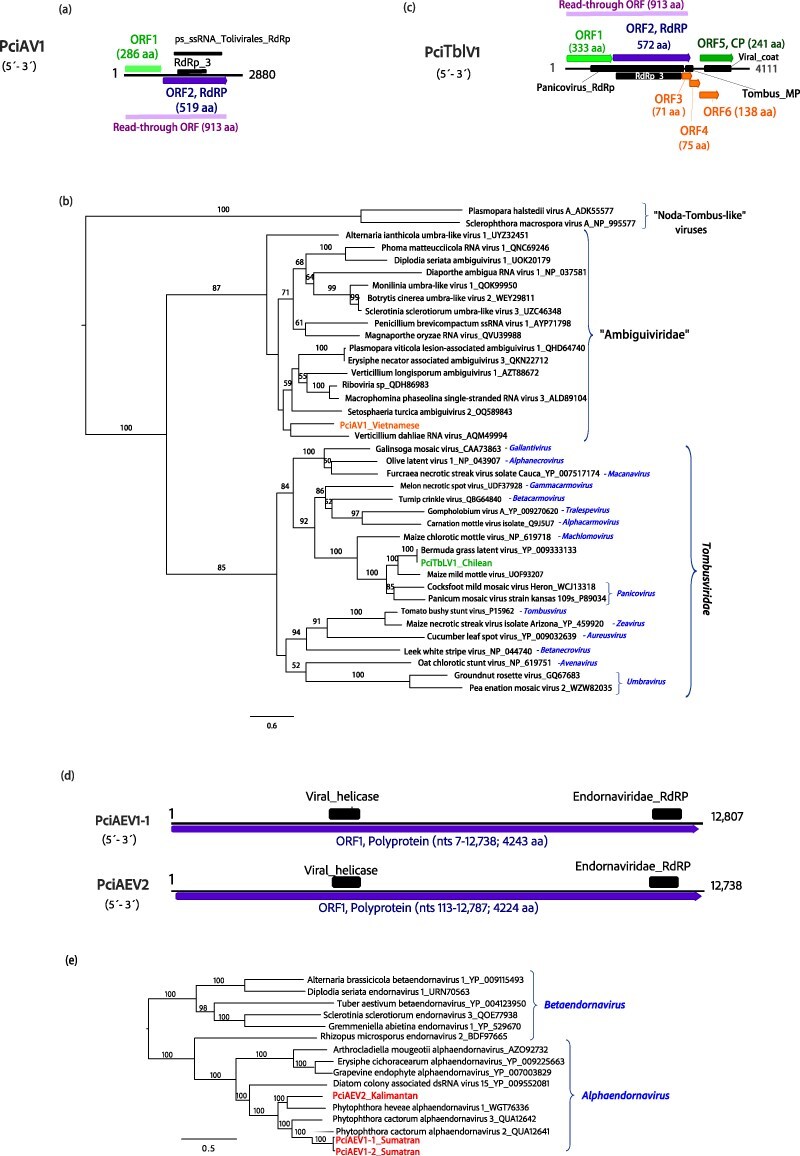
**Description of *P. cinnamomi* (+)ssRNA viruses related to ambiguiviruses, tombusviruses, and endornaviruses. (a)** Graphical representation of Phytophthora cinnamomi ambiguivirus 1 (PciAV1) and Phytophthora cinnamomi tombus-like virus 1 (PciTbLV1). Black boxes represent the conserved domains, colourful boxes represent the ORFs. **(b)** Phylogenetic analysis (RAxML) based on the predicted RdRP of PciAV1 and PciTbLV1 and related viruses of the family *Tombusviridae* and ‘Ambiguiviridae’. **(c)** Graphical representation of Phytophthora cinnamomi alphaendornavirus 1 and 2 (PciAEV1-2). **(d)** RAxML tree depicting the phylogenetic relationships of the predicted RdRP of PciAV1 and 2 with members of the family *Endornaviridae*. In both trees, *P. cinnamomi* viruses appear abbreviated. The geographical origin of viruses is incorporated into their names. Nodes are labelled with bootstrap support values ≥50%. Branch lengths are scaled to the expected underlying number of aa substitutions per site. Tree is rooted in the midpoint. Family classification and the corresponding pBLAST accession numbers are shown next to the virus names. Scale bar shows expected changes per site per branch.

**Figure 3.**
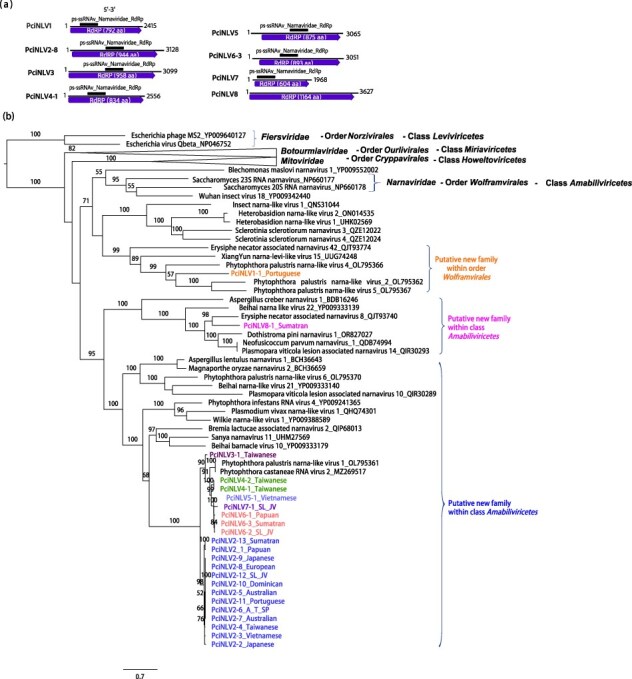
**Description of Phytophthora cinnamomi narna-like viruses. (a)** Graphical representation of Phytophthora cinnamomi narna-like viruses (1-8) (PciNLV1-8). **(b)** Phylogenetic analysis (RAxML) based on the predicted RdRP of narna-like viruses with other complete classified and unclassified members of the phylum *Lenarviricota*. Nodes are labelled with bootstrap support values ≥50%. Branch lengths are scaled to the expected underlying number of aa substitutions per site. Tree is rooted in the midpoint. *P. cinnamomi* viruses are abbreviated and coloured, the variant name and origin of the host are indicated, SL_JV means Sulawesian and/or Javanese origin (P14), ATS means Algerian-Tunisian and/or Spanish origin (P03). Family classification and the corresponding pBLAST accession numbers are shown next to the virus names. Scale bar = 0.9 expected changes per site per branch.

**Figure 4.**
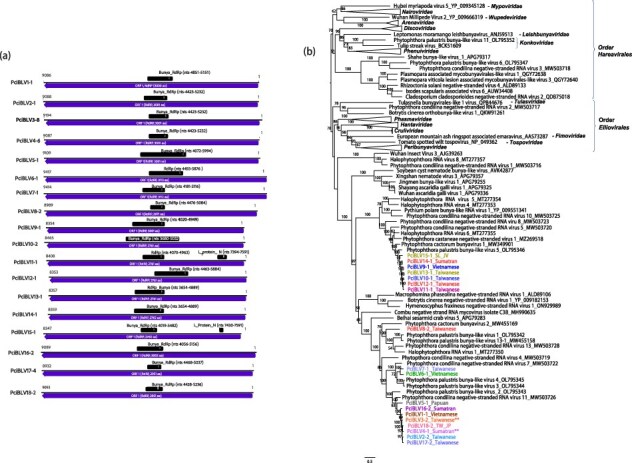
**Description of *P. cinnamomi* viral contigs resembling the L segment of bunya-like viruses. (a)** Graphical representation of one representant of each Phytophthora cinnamomi bunya-like viruses (1-18) (PciBLV1-18). **(b)** Description of (−)ssRNA viruses related to the class *Bunyaviricetes*. RAxML tree illustrating the phylogenetic relationships of the predicted RdRP of bunya-like viruses in *P. cinnamomi* with complete RdRPs of related viruses from the class *Bunyaviricetes*. Some of the classified families appear to simplify the tree. Nodes are annotated with bootstrap support values ≥50%. Branch lengths are proportionally scaled to the anticipated number of aa substitutions per site. Phytophthora cinnamomi bunya-like viruses 18 are denoted by their abbreviated names. The scale bars indicate expected changes per site per branch. Double asterisks represent viruses have an intercontinental distribution. TW_JP: This virus occurs in Taiwan and Japan.

a Acronyms of viral names: Phytophthora cinnamomi ambiguivirus 1 (PciAV1), Phytophthora cinnamomibunya-like viruses 1 to17 (PciBLV1 to 18), Phytophthora cinnamomi chrysovirus 1 (PciCV1) Phytophthora cinnamomi alphaendornaviruses 1 and 2 (PciAEV1 and 2), Phytophthora cinnamomi fusagra-like virus 1 and 2 (PciFLV1 and 2), Phytophthora cinnamomi monormycovirus 1 (PciMOMV1), Phytophthora cinnamomi ormycoviruses 1 to 11 (PciOMV1 to 11)*,* Phytophthora cinnamomi tombus-like virus 1 (PciTLV1), Phytophthora cinnamomi tombus-like virus 1 (PciTbLV1), Phytophthora cinnamomi toti-like viruses 1 and 2 (PciTLV1 and 2).

b Ormycovirid genomic RNA sense is still undetermined.

c SLJV means that this variant appears in Sulawesi, Kalimantan, and/or Java (Indonesia).

d Total reads mapped to the final viral contig sequence.

e number of times each nt in a genome is read during sequencing.

f Conserved domains detected by CDD search; positions in nt sequences are indicated in Table S4 and corresponding figures for each virus type (Figs 1, 2, 3, 4, 6).

g PciMOMV1 putative same virus as PciOMV6.

h At least 60% nt pairwise identity in 5ʹ or 3ʹ end of both segments.

### RNA library preparation and total stranded RNA sequencing

Approximately 1 μg of total RNA, eluted in RNase-free water, was submitted to IABio in Olomouc, Czech Republic, for RNA library construction and deep sequencing. RNA was depleted using the NEBNext rRNA Depletion Kit (Human/Mouse/Rat) and prepared with the NEBNext Ultra II Directional RNA Library Prep Kit for Illumina, along with NEBNext Multiplex Oligos for Illumina (Unique Dual Index Primer Pairs). Library quality control was evaluated with the Agilent Bioanalyzer 2100 High Sensitivity DNA Kit. The KAPA Library Quantification Kit for the Illumina platform facilitated absolute qPCR-based quantification of the Illumina libraries, flanked by the P5 and P7 flow cell oligo sequences. The libraries underwent paired-end (PE) (2 × 150 nt) sequencing on a NovaSeq6000 (DS-150) from Illumina, San Diego, CA, USA, using the NovaSeq S4 v1.5 reagent kit. An ‘in-lane’ PhiX control spike was included in each lane of the flow cell.

### Virus identification bioinformatics pipeline

#### Preprocessing of RNA-seq data

Raw data were automatically processed by BaseSpace cloud interface (Illumina) in default settings. The processing was carried out on the local server of the University of South Bohemia in Česke Budějovice, Czech Republic. The basecalling, adapter clipping, and quality filtering were carried out using Bcl2fastq v2.20.0.422 Conversion Software (Illumina). For the initial quality assessment, we used FastQC v0.11.9 (https://www.bioinformatics.babraham.ac.uk/projects/fastqc/). The adapter sequences, provided by the sequencing company, were verified using BBTools v37.87 (https://jgi.doe.gov/data-and-tools/software-tools/bbtools/) (Bushnell et al. 2017). Subsequent processing with Cutadapt v4.7 (https://cutadapt.readthedocs.io/en/stable/) (Martin 2011) involved trimming of N bases, adapter sequences, and low-quality ends (<30). Reads falling short of 50 bp were discarded from subsequent analyses. Post-trimming quality was again re-assessed with FastQC, revealing overrepresented sequences identified as ribosomal RNA (rRNA) from *P. cinnamomi*. These were effectively removed using SortMeRNA v4.3.6 (https://github.com/sortmerna/sortmerna/) (Kopylova et al. 2012), with the SILVA database of rRNA serving as a reference (https://www.arb-silva.de/).

#### Alignment/read mapping to host genome

For mapping reads to the host genome, the STAR v2.7.9a (Dobin 2014) program was employed with default settings. The NCBI GenBank assembly GCA_018691715.1 (isolate GKB4, South Africa; Engelbrecht et al. 2021) was used as the reference genome for *P. cinnamomi*. After alignment to the reference sequence, all mapped reads were discarded and only unmapped reads were utilized further.

#### Alignment/read mapping to known viruses

To validate the presence of known viruses in the remaining reads, BWA v0.7.17-r1188 (https://bio-bwa.sourceforge.net/) (Li and Durbin, 2010) was used to map the reads to NCBI viral RefSeq sequences at https://www.ncbi.nlm.nih.gov/labs/virus/vssi/#/. The coverage was calculated for each reference virus sequence by SAMtools v1.16.1 (https://github.com/samtools/samtools/) (Li et al. 2009). For sequences where the coverage exceeded 80%, alignment visualization was carried out using IGV v2.16.1 (https://software.broadinstitute.org/software/igv/) (Robinson et al. 2011).

#### De novo assembly of unmapped reads for detection of novel viruses

The unmapped reads were used for de novo assembly. To assemble these reads into contigs, the SPAdes program v3.15.5 (https://github.com/ablab/spades/) (Bankevich et al. 2012) was used with default settings for metagenomics. For further analysis, assembled contigs shorter than 500 bp were discarded.

#### Search for similarity in virus databases

Final contigs were cross-referenced with several databases, contigs using the Basic Local Alignment Search Tool (BLAST) v2.10.0 (https://blast.ncbi.nlm.nih.gov/doc/blast-help/downloadblastdata.html/ (Camacho et al. 2009). As a reference, we used a list of viral reference sequences from NCBI. BLASTn was applied for the nucleotide (nt) database accessible at https://www.ncbi.nlm.nih.gov/labs/virus/vssi/#/virus?SeqType_s=Nucleotide. Similarly, BLASTx was employed for the protein database available at https://www.ncbi.nlm.nih.gov/labs/virus/vssi/#/virus?SeqType_s=Protein, and also to search the UniProt database for a specified virus taxon at https://www.uniprot.org/uniprot/?query=taxonomy:10239. Contigs with e-value higher than 1e-3 were discarded. Subsequently, the remaining contigs were processed in a similar manner using the entire range of databases. For BLASTn, the nt database from NCBI, available at https://ftp.ncbi.nlm.nih.gov/blast/db/nt*, was used. For BLASTx, both the nr database from NCBI accessible at https://ftp.ncbi.nlm.nih.gov/blast/db/nr* and UniProt Knowledgebase (UniProtKB) available at https://www.uniprot.org/uniprotkb/, were employed. The BLAST search was restricted to the best hit per contig. The final list of candidate viral contigs was compiled by scanning the BLAST results for the search string ‘vir’. Next, final contigs with certain similarity to viruses available in the GenBank were classified into taxonomic groups and performed pairwise alignments of both the nt sequences and their corresponding amino acid (aa) sequences to define potential species and variants (see section ‘Criteria for defining distinct viruses and variants’).

#### Identification of potential ORFan viruses

The ORFan analysis was conducted similarly to the studies by Chiapello et al. (2020) and Forgia et al. (2022) with a few modifications. Briefly, in the first step, contigs with a length of <1000 bp were discarded. After the filtration, open reading frames (ORFs) within the remaining contigs were identified utilizing ORFfinder v0.4.3 (Rombel et al. 2002). The minimum length of ORFs was set at 100 aa. Next, the filtered set of ORF-containing contigs underwent read mapping using BWA v0.7.17-r1188. Based on the alignment, only contigs exhibiting a minimum coverage of 95% and an average sequencing depth exceeding 10× in both forward and reverse directions were left for further evaluation. In the last step, the contigs were subjected to a homology search against the nr NCBI database using the BLASTx. Only contigs without any hits were considered as potential novel virus fragments.

### Clustering’s analyses of the ORFan sequences

To explore similar contigs in different pools, clustering’s analyses were conducted on the ORFan contigs from previous analyses. Initially, the analysis was carried out using nts sequences of these contigs, second with the aa sequences obtained by translating the identified ORFs.

For the clustering of nt sequences of the contigs, the ALFATClust tool was used (https://github.com/phglab/ALFATClust) (Chiu and Ong 2022). Only clusters that included at least two sequences, each originating from distinct pools, were kept. Each cluster was subjected to multiple sequence alignment (MSA) using the Clustal Omega aligner (Sievers et al. 2011). The results of the MSA were carefully examined through visual inspection to validate the quality of clustering.

In parallel, aa sequence clustering was performed in a similar manner. The process began with the identification of ORFs within the contigs using the ORFfinder tool (https://www.ncbi.nlm.nih.gov/orffinder/) (Rombel et al. 2002), followed by the translation of these ORFs into aa sequences. These sequences were then analyzed for similarity with the nr database of NCBI using BLASTp. Any aa sequences with positive hits identifying known proteins were excluded from the next step. The remaining sequences were clustered using the ALFATClust tool (Chiu and Ong 2022), and alignments were conducted using the Clustal Omega similar to the nt sequence analysis (Sievers et al. 2011). The MSA outcomes were visually examined to validate the quality of clustering. Clusters that contained at least two aligned sequences, each from different pools, were considered as potential new virus fragments. In the final step, these candidate sequences were analysed for similarity using the local InterProScan tool (https://ftp.ebi.ac.uk/pub/software/unix/iprscan/5/5.67-99.0/interproscan-5.67-99.0-64-bit.tar.gz) (Blum et al. 2021).

### Coverage depth

The number of unique sequencing reads that align to each reference de novo assembled viral contig was calculated with the following formula: (Total reads mapped to the final identified virus × average read length)/virus genome or contig length) (Sims et al. 2014). Coverage plots were visualized with Integrated Genome Viewer (IGV) tool and Geneious Prime® 2025.0.2.

### Genetic variability analyses and conserved domains

Nucleotide (nt) and aa sequences were aligned using MAFFT (Biomatters Ltd v1.4.0) (Katoh et al. 2002), and pairwise sequence comparison identities were calculated in Geneious Prime® 2021.0.4. This program was also used for primer design. To identify conserved domains within the putative viral proteins, the NCBI CDD-search tool was employed (https://www.ncbi.nlm.nih.gov/Structure/cdd/wrpsb.cgi).

### Phylogenetic analyses

Maximum likelihood (ML) phylogenetic trees were generated using a rapid bootstrapping algorithm in RAxML-HPC v.8 on XSEDE through the CIPRES Science Gateway (Stamatakis 2006). The tree search was conducted under the GAMMA model to prevent extensive optimization of the best scoring ML tree at the run’s conclusion. The Jones–Taylor–Thornton (JTT) model was selected as the substitution model for proteins. Bootstrapping was configured with the recommended parameters provided by CIPRES Science Gateway. The resulting data were visualized using FIGTREE version 1.4.4 software.

#### Criteria for defining distinct viruses and variants

To differentiate between distinct viruses (putative species), we used the criteria of nt and aa identity specified for each type by the International Committee on Taxonomy of Viruses (ICTV), i.e. <70% or <90% identity, depending on the viral taxa (see results section). Viruses should also form separate, well-supported clades in phylogenetic trees (bootstrap support ≥ 70%). Variants of the same virus were defined based on nt and aa divergence being within the expected virus threshold.

### RT-PCR detection of virus presence in individual host isolates

The presence of each identified virus was validated through direct reverse transcription polymerase chain reaction (RT-PCR) using total RNA as a template and primers specifically designed for viruses and/or specific variants. The High-Capacity cDNA Reverse Transcription Kit (Applied Biosciences, Park Ave, NY, USA) was employed for cDNA synthesis. PCRs were conducted using Hot Start Taq 2× Master Mix (New England BioLabs, Ipswich, MA, USA), comprising 25 µL Master Mix, 1 µL of each primer (10 mM), and 4 µL of cDNA in a total volume of 50 µL. RT-PCR products were visualized through gel electrophoresis (120 V; 60 min) on a 1.5% agarose gel prepared with TBE 1X buffer (Merck KGaA, Gernsheim, Germany) and stained with Ethidium bromide (SIGMA–Aldrich, Steinheim, Germany). For discriminant analysis of principal components (DAPC) common suitable primers for amplifying variants were used (see Table S3). PCR products displaying the expected amplicon lengths were purified and sequenced by GATC BioTech (Eurofins; Konstanz, Germany) in both directions using the primers employed for PCR amplification. The primers for the partial amplification of the RNA-dependent RNA polymerase (RdRP) of each virus (Table S2) were designed using Primer 3 2.3.7 under Geneious Prime® 2020.0.4. Amplicon sequences can be found here in this link: 10.6084/m9.figshare.26029246.

### Statistics, graphs, and visualizations

Maps and graphs were created based on physically measured GPS coordinates using the R version 4.0.5 (https://www.R-project.org/) R Core Team, (2023) programming environment using the special libraries ggplot2, sf, mapplots, ggspatial, scatterpie, and ggrepel.

The sequence data were processed with the ape package (Paradis and Schliep 2019) and the adegenet package (Jombart 2008; Jombart and Ahmed 2011) using one-hot-encoding, where four dummy variables were created for each nt position, representing the presence/absence of four bases. This approach allowed to account for IUPAC ambiguity characters. Gaps were coded as four zeroes—the absence of all the nts. To discriminate between populations from different continents and countries, assess differences between them, and identify clusters of genetically related individuals, DAPC analysis was performed using the package adegenet (Jombart 2008; Jombart and Ahmed 2011). The number of axes retained in the principal component analysis step was set to 30. The number of axes retained in the discriminant analysis step was set to the number of populations minus one. To statistically evaluate each individual comparison, the analysis of molecular variance (AMOVA) was carried out using the package poppr (Kamvar et al. 2014, 2015) followed by the permutation test implemented in the randtest function from the ade4 package (Dray and Dufour 2007) with 999 permutations. Only those populations for which at least five samples were available were included in this analysis. The Benjamini–Hochberg correction for multiple comparisons (Benjamini and Hochberg 1995) was applied. The DAPC scatter plots were visualised using the packages ggplot2 (Wickham 2016), ggrepel (Slowikowski 2023), RColorBrewer (Neuwirth 2022), patchwork (Pedersen 2022), and svglite (Wickham et al. 2023a). The package dplyr (Wickham et al. 2023b) was used for common data cleaning tasks.

### Estimation of the 3D-protein structure

Structural models of ormycovirid hypothetical proteins (HPs) were built using AlphaFold2 through the colab fold (Mirdita et al. 2022) with standard settings. Structure models were compared pairwise with UCSF ChimeraX (https://pubmed.ncbi.nlm.nih.gov/32881101/) showing the pLDDT value obtained for each model and the RMSD value for each pairwise comparison (calculated both on the full-length sequence of the proteins and on the conserved portion).

## Results

### Virus identification, description, and classification

#### RNA-seq results

A total of 21 sequenced RNA libraries produced from 222 isolates of *P. cinnamomi* (Table S1) generated a total of 18 348 614 166 PE reads, out of which, at least 29 083 643 reads appeared to be viral. The pool with the highest viral read amount was pool (P) 01 with 3 292 400 PE reads, and the pool with the lowest viral read number was P08 with 336 598 PE reads (Table S3). All RNA-sequencing results are shown in Supplementary Table S2. Sequence Read Archive (SRA) records are accessible in the bioproject PRJNA1102859 using the link https://www.ncbi.nlm.nih.gov/sra/PRJNA1102859, and GenBank accession numbers PP891625–PP891946 corresponding to the final viral sequences described in this study are listed in Tables 1 and S4.

#### dsRNA viruses

Derived from pool 15, a virus resembling members of the *Chrysoviridae* family (phylum *Duplornaviricota*; class *Chrymotiviricetes;* order *Ghabrivirales;* suborder *Alphatotivirineae*) was identified from 18 final contigs (Tables 1 and S4). This virus exhibits a multipartite genome with four distinct monocistronic linear dsRNA segments, characteristic of the *Alphachrysovirus* genus. Specifically, dsRNA1 encodes the RdRP (1115 aa), dsRNA2 encodes the coat protein (CP, 998 aa), dsRNA3 encodes a hypothetical protein (P3, 887 aa), and dsRNA4 encodes a protease (P4, 806 aa) (Fig. 1a). Although random amplification of cDNA ends (RACE) was not conducted, the presence of highly similar sequences in all four 5 ʹ-UTRs might suggest their completeness in this terminus (Fig. 1b). Furthermore, each dsRNA segment contains 3–4 ‘CAA’ repeats (not shown), consistent with the type members of the *Alphachrysovirus* genus. Thus, this virus is designated as Phytophthora cinnamomi chrysovirus 1 (PciCV1), marking the first chrysovirus described in a *Phytophthora* species. Phylogenetic analysis of PciACV1’s RdRP with other members of the *Chrysoviridae* family confirms its taxonomic position within the *Alphachrysovirus* genus, showing a close relationship with *Alphachrysovirus aspergilli* (Fig. 1c).

RT-PCR screening confirmed the presence of PciACV1 in a single *P. cinnamomi* MAT A1 isolate in Sumatra (Figure S1).

Three fusagravirids (phylum *Duplornaviricota*; order *Ghabrivirales;* suborder *Alphatotivirineae*; family *Fusagraviridae*) with length range from ∼6.9 to 7 kb were identified through the assembly of 42 contigs exceeding 500 nts in pools 7, 13, and 1. Considering both pairwise sequence identity comparison (Tables S5a, b,c) and species demarcation criteria 3 and 4 of the last 2023.015 F-ICTV proposal for the order *Ghabrivirales* (<70% RdRP aa sequence identity), they were designated as Phytophthora cinnamomi fusagra-like virus 1 and 2 (PciFLV1 and 2). Thus, PciFLV1 has two variants, also confirmed by RT-PCR, one detected in one Vietnamese MAT A2 isolate and another detected in two Sumatran MAT A1 isolates. PciFLV2 was detected in one Sumatran MAT A1 isolate, Figure S1. All three viruses comprise two discontinuous ORFs, with ORF1 (5ʹ-terminus proximal ORF) encoding the putative CP and ORF2 (3ʹ-terminus proximal ORF) encoding the RdRP. Conserved motifs of the RdRP_4 family (PF02123) were identified in all three ORF2 sequences (Fig. 1d; Table 1). The three genomes have a heptamer immediately upstream of the termination codon of ORF1 at map positions (see Fig. 1d).

In the phylogenetic tree (Fig. 1c), PciFLV1 and 2 form a cluster with a virus discovered in the arthropod *Ochlerotatus* in Finland, while PciFLV2 clusters with other fusagraviruses detected in *Phytophthora* species.

Upon BLASTx results, two more viruses related to the order *Ghabrivirales* were detected in pools 07 and 14 (Tables 1 and S4). One of them resembled members of the recently approved family *Ootiviridae* (phylum *Duplornaviricota*; class *Chrymotiviricetes;* order *Ghabrivirales*; suborder *Betatotivirineae*). The other one appears to be related to unclassified toti-like viruses potentially belonging to a new family within the suborder *Alphatotivirineae* (Fig. 1c). The alignment of these contigs revealed an aa pairwise identity of 37.89%, indicating that they represent two different toti-like viruses (species), designated as Phytophthora cinnamomi toti-like viruses 1 and 2 (PciTLV1 and 2). Each contig spans ∼5.4–6.3 kb and contains two ORFs when translated using the standard translation Table 1. Notably, conserved domains belonging to the RdRP_4 superfamily member pfam02123 were identified in the 3ʹ-terminus proximal ORF of both contigs (Fig. 1e; Table 1). However, no conserved domains of the totivirus coat superfamily, cl25797, and pfam05518, were detected in the 5ʹ-terminus proximal ORF. Given the genomic organization similar to that of members of both suborders *Beta*- and *Alphatotivirineae*, it is proposed that ORF1 (5ʹ-proximal) encodes the capsid protein (Gag). Notably, the genomic features of PciTLV1 and 2 exhibit slight differences. Specifically, PciTLV1 lacks overlapping ORFs observed in PciTLV2 by 4 nts (CTGA). Upon utilizing the stop-codon read-through translation table, a longer ORF encoding a Gag-Pol protein (1108 aa) is detected in PciTLV2 from nt 3420, where an AUG initiator is located. Interestingly, these observations are not evident in PciTLV1, which features a shorter ORF1 and longer 5ʹ-untranslated regions (UTR) (956 nts). No alternative initiation (non-AUG codons) was detected in either virus.

The RT-PCR screening confirmed that PciTLV1 is hosted by four Vietnamese MAT A1 and three MAT A2 isolates of *P. cinnamomi* (44% of the isolates in pool P07), which were all collected in the Hoang-Lien Mountains, northern Vietnam, Table S1). In contrast, PciTLV2 is only hosted by one Sulawesian MAT A1 isolate (Figure S1).

#### (+)ssRNA viruses

Five final contigs derived from the de novo-assembling had affinities with ambiguiviruses, (phylum *Kitrinoviricota;* class *Tolucaviricetes*; order *Tolivirales*) in pool 07. These contigs were identified as originating from the same virus, subsequently named Phytophthora cinnamomi ambiguivirus 1 (PciAV1). With a genome length of 2880 nts (Fig. 2a; Table 1), PciAV1 contains two ORFs when translated using standard translation Table 1. ORF 1 encodes an HP lacking classified conserved domains, while ORF 2 codes for the RdRP and encompasses conserved regions of the Tolivirales_RdRp (cd23179) and RdRP_3 (pfam00998) families. When using the stop-codon read-through translation table, ORF1 and 2 become one single ORF with a length of 2487 nts and 829 aa. Phylogenetically, PciAV1 is closely related to Verticillium dahliae RNA virus (Fig. 2b). Notably, this virus is hosted by a single Vietnamese MAT A2 isolate of *P. cinnamomi* (Figure S1).

In the RNA library/pool 01, five contigs displaying 100% identity were identified from *P. cinnamomi* isolates collected in Chile (Tables 1, S4 and Figure S2). The final viral contig exhibited a length of 4111 nts and shared 99% identity with Bermuda grass latent virus (BGLV), potential member of the genus *Panicovirus* (phylum *Kitrinoviricota;* class *Tolucaviricetes;* order *Tolivirales*). Additionally, this virus BGLV (NC_03240) had been detected in this pool when applying the bioinformatics pipeline for detection of known viruses with a percentage coverage of 99.85 and mean depth of 110. Designated as Phytophthora cinnamomi tombus-like virus 1 (PciTbLV1), its genome comprises six ORFs: ORF1 encoding a potential replicase-associated protein; ORF2 encoding the RdRP; ORF3 and 4 encoding small mobile proteins (MP); ORF5 encoding the coat protein; and ORF6, which is imprinted in ORF5 and might be an accessory MP (Fig. 2c). Conserved motifs of the Panicovirus_RdRp (cd23238) and RdRP_3 (pfam00998) families are present, alongside motifs of the Tombus_movement super family (cl05068) and the viral coat protein (PF00286). Additionally, when utilizing the stop-codon read-through translation table, ORF1 and ORF2 merge into a single ORF with a length of 2739 nts and 913 aa. Phylogenetic analyses place PciTbLV1 conclusively within the *Tombusviridae* family, clustering with the plant pathogen BGLV (Fig. 2b). Interestingly, PciTbLV1 was confirmed in two out of eight *P. cinnamomi* isolates collected in Chile (Figure S1). The occurrence of this virus in *P. cinnamomi* suggests a cross-kingdom transmission event.

BLASTx results showed that in pools 13 and 15 (consisting of RNA from Sumatran isolates), a total of six contigs longer than 500 nts were discovered, displaying affinities with endornaviruses of the family *Alphaendornaviridae* (phylum *Kitrinoviricota*; class *Alsuviricetes*; order *Martellivirales*) (Table 1). Similarly, pool 14 (comprising pooled RNA from Sulawesian, Javanese and Kalimantan isolates) yielded 26 contigs with the same endornavirus characteristics (Fig. 2d). Following the removal of redundant sequences, three prominent endornavirids emerged, measuring 12 807, 12 813 and 12 843  nts in length, respectively (Table 1; Table S4). The three endornavirids feature a large positively framed ORF encoding a polyprotein and around 40% GC content. The three-sequence alignment revealed 5855 nt identical sites (56.9% pairwise identity) and 1664 identical sites in aa sequences (37.6% pairwise identity). According to the species demarcation criteria set by the ICTV for the genus *Alphaendornavirus*, viruses must exhibit an overall nt sequence identity below 75.0% to be classified as members of two distinct species. Therefore, the three endornaviruses were designated as Phytophthora cinnamomi alphaendornavirus 1 and 2 (PciAEV1 and 2) with PciAEV1 having two variants (PciAEV1-1, 1-2) (individual pairwise differences are shown in Table S6 and phylogenetic relationships in Fig. 2e). Both variants 1 and 2 of PciAEV1’s polyprotein consist of 4243 aa with a molecular weight of, respectively, 480.967 kDa and 481.058 kDa. PciAEV2 encodes a polyprotein of 4224 aa, weighing 479.566 kDa. Notably, conserved motifs of Endornaviridae_RdRp (cl40470), RdRP_2 (pfam00978), and Viral (Superfamily) RNA helicase (PF01443) were identified in both genomes (Table 1).

Subsequent screening via RT-PCR confirmed the presence of both endornaviruses exclusively in Indonesian MAT A2 isolates. Specifically, PciAEV1 was detected in two isolates from Sumatra, while PciAEV2 was found in a single Kalimantan isolate (Figure S1).

Virus contigs resembling members of the family *Narnaviridae* (phylum *Lenarviricota*; class *Amabiliviricetes*; order *Wolframvirales*) were present in most pools, except for pool 01 (Chile) (Figure S1; Table S1). Following the removal of redundant contigs and the selection of the longest ones containing an ORF, a total of 23 narnaviral sequences were identified (Tables 1, S4). These sequences exhibit genomic features ranging from ∼2 to 3.7 kb in length, each containing a unique positively framed ORF encoding the RdRP. The majority of contigs exhibit conserved domains typical of RdRP in *Amabiliviricetes* class (Fig. 3a; Table 1). A MAFFT alignment of both nt sequences and aa RdRP sequences of the 23 narna-like contigs was conducted to calculate the PASC percentages (Table S7). The overall nt and aa pairwise sequence similarity was relatively high (71.6% and 70.1%, respectively), but based on the species demarcation criteria proposed by the ICTV for the family *Narnaviridae*, eight distinct viruses were identified and designated as Phytophthora cinnamomi narna-like viruses 1-8 (PciNLV1-8).

The phylogenetic analyses, based on the aa sequences of their RdRP (Fig. 3b and S3), revealed that PciNLV1-8 are clustered into three distinct groups, which could be considered three potential new families. One of them, within the order *Wolframvirales*, would contain PciNLV1, which was identified in Portuguese isolates, and exhibits close relationships to Phytophthora palustris narna-like virus 2, 4, and 5 (PpaNLV2, 4, and 5). Another family would include PciNLV2 (including all 13 variants), 3, 4 (with 2 variants), 5, and 6 (featuring 3 variants), which are closely associated with each other and with Phytophthora palustris narna-like virus 1 (PpaNLV1) and Phytophthora castaneae RNA virus 2 (PcaRV2). And, the last family would contain PciNLV8 which appears to have a closer affinity to a narnavirus discovered in *Erysiphe necator*-associated plant lesions. These last two families might belong to a possible new order within the class *Amabiliviricetes*.

PciNLV2 exhibits a global distribution, being identified on every continent screened in this study except South America (Figure S1). Conversely, PciNLV3, 4, 5, 6, 7, and 8 are exclusively found in Southeast Asia.

#### (-)ssRNA viruses

A total of 51 viral contigs resembled with the L segment (RdRP-encoding segment) of putative members of the extensive class *Bunyaviricites* (phylum *Negarnaviricota*, subphylum *Polyploviricotina*) according to BLASTX search (Tables 1, S4). Based on the PASC of the aa and nt sequences (Tables S8 and S9) and considering pairwise aa and nt identity >90% and the phylogenetic relatedness as criteria to belong to the same virus, a total of 18 putative viruses were identified together with their respective variants. They were named as Phytophthora cinnamomi bunya-like viruses 1-18 (PciBLV1-18). All the bunya-like contigs contain a single large ORF (Fig. 4a). In all of them conserved domains (CDD) of Bunya_RdRP were detected. And, even in PciBLV11-1 and PciBLV15-1, the CDD of Bunya_L_protein_N_terminus; pfam04196 was also found (Fig. 4a, Table S4). Across all libraries, we did not detect any additional orphan clusters that could correspond to other genomic segments of the described bunyaviruses. Based on termini sequences of the L segments, a search of similar conserved terminal ends among the orphan sequences did not find evidence of conserved termini corresponding to additional segments.

Phylogenetic analyses were conducted to confirm the identification of 18 viruses (Fig. 4). The RAxML tree was constructed using the 51 viral RdRP sequences (Table S4), revealing distinct clusters (Figure S4). Notably, one cluster encompassed PciBLV9, 10, 11, 12, 13, 14, and 15, while another significant cluster included PciBLV1, 2, 3, 4, 5, 16, 17, and 18. Additional smaller clusters emerged, containing PciBLV5, 6, 7, and 8. In general, each virus appears to be confined to a specific Asian country (or an island within a country). However, PciBLV3 and 4 exhibit an international distribution.

### Novel bisegmented and putative monosegmented RNA viruses

**Figure 5.**
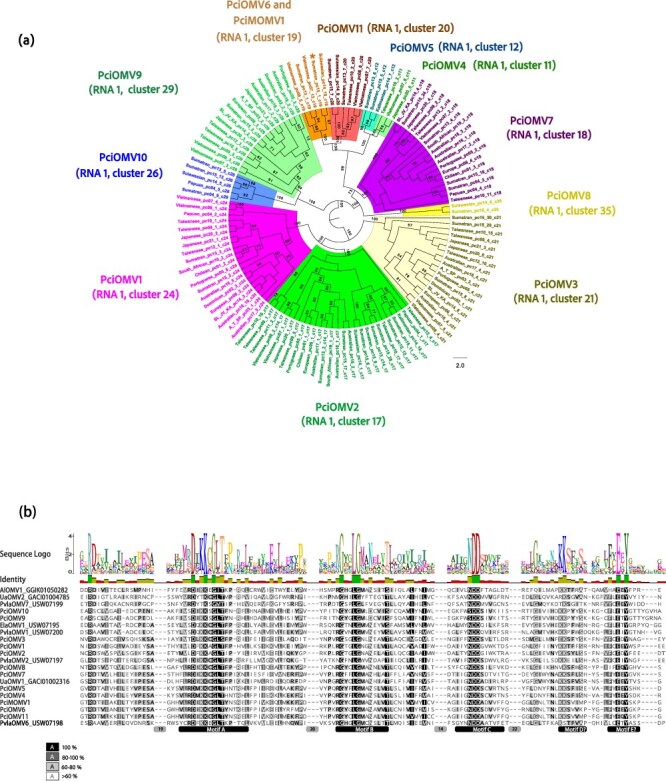
**(a)** Circular RAxML cladogram tree based on the predicted RdRP of *P. cinnamomi* ormycoviruses’ contigs (PciOMV1-11). Species and variants are colour-coded. Variants names include the country of origin of *P. cinnamomi* host, the RNA pool number where they were detected (pc) and the cluster of nt and protein similarity percentage initially assigned, necessary for the species description. More information can be found in Table 1 and Table S3. SL_JV_KA means the variant was found in the RNA pool with Kalimantan, Sulawesian, and Javanese origin; A_T_SP means Algerian–Tunisian and/or Spanish origin. **(b)** Amino acid alignment of the putative conserved domains of the RdRP proteins encoded by *P. cinnamomi*’s ormycoviruses and PvlaOMV2 (Plasmopara viticola lesion-associated ormycovirus 2_USW07197), UaOMV2 (Uromyces appendiculatus ormycovirus 2_GACI01004785), PlvlaOMV2 (Plasmopara viticola lesion-associated ormycovirus 7_USW07199), ElaOMV1 (Erysiphe lesion-associated ormycovirus 1_USW07195), PlvlaOMV1 (Plasmopara viticola lesion-associated ormycovirus 1_USW07200), AlOMV1 (Ambispora leptoticha ormycovirus 1_GGIK01050282), UaOMV1 (Uromyces appendiculatus ormycovirus 1_GACI01002316), and PvlaOMV6 (Plasmopara viticola lesion-associated ormycovirus 6_USW07198). Conserved residues from RdRP palm-domain motifs A, B, and C, and potential motifs D and E are indicated in black boxes. Lower numbers show the deleted positions on the alignment. The percentage of similarities is calculated based on Blosum62 score matrix with a threshold of 1. Sequence logos represent the conservation of aa (in protein sequences). The relative sizes of the letters indicate their frequency in the sequences. The total height of the letters depicts the information content of the position, in bits.

**Figure 6.**
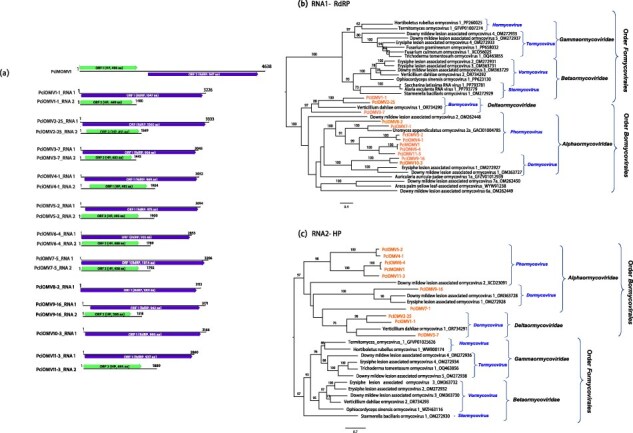
**Description of the ormycoviruses found in *P. cinnamomi*. (a)** Illustration of the genome of *P. cinnamomi*’s ormycoviruses. **(b)** RAxML tree showing the phylogenetic relationships of the RdRPs (RNA1) and HPs (RNA2) **(c)** of *P. cinnamomi*’s ormycovirus species (only one variant selected) with other reported members of the class *Orpoviricetes*. Nodes are labelled with bootstrap support values ≥50%. Branch lengths are scaled to the expected underlying number of aa substitutions per site. Tree is rooted in the midpoint. Scale bar represents expected changes per site per branch.

A total of 274 conclusive contigs were identified within the genetic compositions of ‘ormycoviruses’, a novel category of bisegmented RNA viruses distinct from all known *Riboviria* members first documented by Forgia et al. (2022) and proposed as a new class under the name *Orpoviricetes* (Botella et al. ICTV proposal: 2024.008 F.Uc.v2.Orpoviricetes_newclass.docx). These sequences were initially assembled and identified using the ‘orfan’ pipeline detailed above, subsequently clustered based on shared nt and aa sequences across all RNA-seq pools, and screened for the presence of ORFs and alternative Palm codons. Specifically, 123 nt sequences, each with a common length of 3–3.3 kb, were assigned to RNA1 segments containing the putative RdRP (Fig. 5a). Additionally, 151 sequences, ranging from 0.6 to 1.9 kb in length, were matched to the RNA2 segment, which encodes an HP (Figure S5). Furthermore, a single sequence ∼5 kb in length was identified as a bicistronic RNA (Fig. 6a), encoding both RdRP and HP. The reconstruction of their phylogenetic relationships (Fig. 5a) and the pairwise identity comparisons (Tables S10 and S11) supported the occurrence of 11 separated clusters, which eventually represented putative 11 virus species (and their variants). They were designated as Phytophthora cinnamomi ormycoviruses 1-11 (PciOMV1-11) and Phytophthora monormycovirus 1 (PciMOMV1). Detailed information on all these viruses is given in Table S4, and one representative variant of each species is included in Table 1. Likewise, RNA2 segments were identified through the 21 pools and assigned to each RNA1, with the exception of PciOMV8 and 10 (Fig. 6a). This assignment was based on common presence of RNA1 and RNA2 in pools and individual host isolates by RT-PCR, contigs’ termini-similarity, RNA1/RNA2 mapped reads ratio, and phylogenetic relationships among each other. The putative monosegmented PciMOMV1 encloses the two ORFs resembling those encountered in PciOMV6, the RdRP groups in cluster 19, and the HP joins cluster 32 (Fig. 6a). It was only detected in two isolates in the Sumatran pool 15, confirmed through primer design and PCR targeting the putative intergenic space, which has an extremely low read coverage (Figure S7; see amplicon sequences in 10.6084/m9.figshare.26029246/). No circularity was confirmed when using primers designed at 5ʹ and 3ʹ ends and no ribozymes were detected *in silico* in this contig.

The conservation of the aa sequence seems to be considerably higher in RdRPs than in the HPs. The MAFFT alignment of the RdRPs enabled the recognition of certain conserved regions in the A–B–C motifs of the Palm catalytic domain (Fig. 5b). Thus, the presence of the aa residues serine (S), aspartate (D), and aspartate (D) within motif C was found in PciOMV9 and 10, while all the rest had asparagine (N), aspartate (D), and aspartate (D). Identity matrix built on MAFFT alignment of the HPs show low conservation among the putative proteins (Figure S6). Significant identity is observed only inside the groups identified through phylogenetic analysis on the RdRP (Fig. 6b and S5). To observe structural conservation between HPs without identity at the aa level (thus potentially suggesting functional conservation) structural models were built from each of the identified HPs comparing them to detect conservation through UCSF ChimeraX. For most of the HPs, it was impossible to detect a good model (pLDDT values higher that 70). Pairwise comparison between models with good pLDDT values showed good structural conservation among proteins demonstrating higher identity on the identity matrix (Figure S5a and b) while poor conservation is observed by comparing HPs from distant viruses (PciMOMV1 and PciOMV1 in Figure S5).

The RAxML analyses, reconstructing the phylogenetic relations between the RdRPs and the HPs of PciOMV1-11, PciMOMV1, and known ormycoviruses, indicate that they are distributed in two families, *Deltaormycoviridae* and *Alphaormycoviridae*, belonging to the order *Bormycovirales* (2024.008 F. Uc.v2.Orpoviricetes_newclass.docx) (Fig. 6b, c). Ormycoviruses have their highest abundance and diversity in Indonesia, in particular Sumatra, where the 11 species occur (Fig. 5a, S1).

### Viral abundance and diversity differences across continents and regions

**Figure 7.**
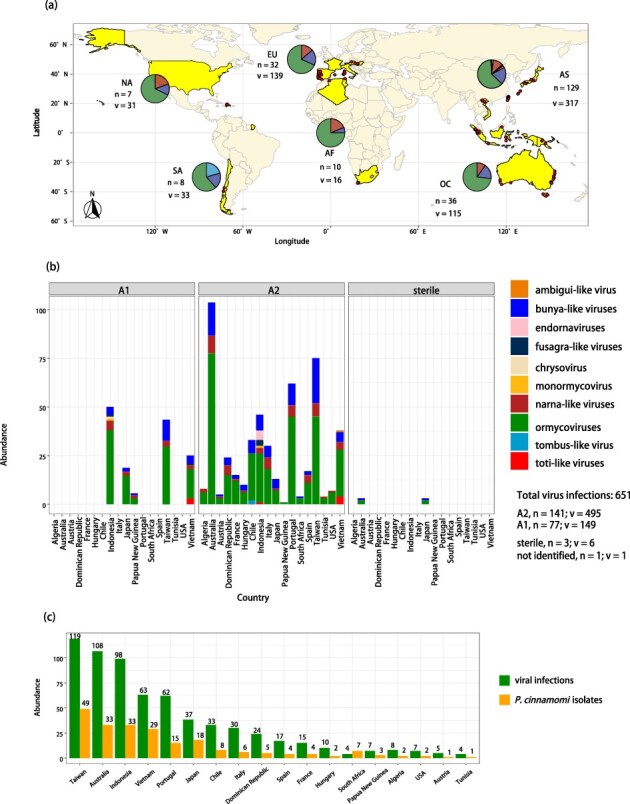
**Distribution and abundance of viruses in a global collection of *P. cinnamomi*. (a)** World map showing the sites of the virus-hosting *P. cinnamomi* isolates (more information in Table S1). The pie charts illustrate the relative frequency of each virus types (family/order) in Africa (AF), Asia (AS), Europe (EU), North America (NA), Oceania (OC) and South America (SA) considering the total occurrence and number of *P. cinnamomi* isolates (n) and virus infections (v). **(b)** Bar charts showing the overall abundance of virus types considering mating types MAT A1 and MAT A2 and country. **(c)** Bar charts showing the overall abundance of viruses in relation with the number of isolates.

The RT-PCR screening of viruses throughout all 222 *P. cinnamomi* isolates from all regions (countries) and continents resulted in 651 distinct viral infections derived from 46 viruses (species). Among them, ormycovirid infections are most abundant (440 infections, 68%), followed by bunyavirids (119 infections, 18%), narnavirids (70 infections, 11%), and, with lower proportions, totivirids (8 infections, 1%), fusagravirids (5 infections, 1%), endornavirids (5 infections, 1%), tombusvirids (2 infections) chrysovirid and ambiguivirid (1 infection each). Ormycovirids and bunyavirids occur in all continents and countries (Fig. 6a) whereas narnavirids are only absent from South America (Chile). All other viral types only occur in Asia (Fig. 7a, b). Both the absolute highest virus diversity and abundance occur in Asia (45 virus species = 95.7%, 129 isolates, 317 infections, 2.46 infections per isolate), followed by Europe (9 virus species, 32 isolates, 139 infections, 4.34 infections per isolate), Oceania (11 virus species, 36 isolates, 115 infections, 3.19 infections per isolate), South America (5 virus species, 8 isolates, 33 infections, 4.13 infections per isolate), North America (5 virus species, 7 isolates, 31 infections, 4.43 infections per isolate), and Africa (8 virus species, 10 isolates, 16 infections, 1.6 infections per isolate). At regional scale (Fig. 6b), the average ratio of virus infections per isolate is the highest in Austria, Hungary, and Italy, followed in a decreasing order by Dominican Republic, Portugal, Chile, Australia, France, USA, Spain, Tunisia, Algeria, Indonesia, Taiwan, Vietnam, Japan, Papua New Guinea, and South Africa (Fig. 7c). However, the highest taxonomic diversity (range of virus families, genera or broader taxonomic groups, and species represented) is observed in Indonesia (8 higher phyla and 26 species) and subsequently Vietnam (6 higher phyla and 16 species) (Fig. 7b; Table S4). While Taiwan exhibits the highest abundance, its viral diversity appears to be consistent with other regions worldwide, including Japan, Australia, and Portugal. Remarkably, the *P. cinnamomi* population in Chile constitutes a distinct case, characterized by the exclusive infection of a unique tombusvirus not observed elsewhere.

Of the 222 *P. cinnamomi* isolates, 80% harbour at least one virus, leaving only 20% without any viral presence. Further examination reveals that 13% of the isolates solely host a single virus, while a majority of 67% exhibit double or multiple virus infections, indicating a complex interplay between viral pathogens and their hosts. Particularly noteworthy is isolate VN0188 (MAT A1), which hosts 12 distinct viral species, the highest number recorded among all isolates screened. Other Asian isolates (A2) show at least ten (VN0236, VN0210, and KA0399) or eight (TW0287 (A2) and SL0204 (A1)) viral infections. Single infections only occur in Asia and Papua New Guinea (Oceania), while in the rest of the world, only double or multiple infections or—less frequently—no infections occur (Figure S1).

### Viral abundance and diversity differences between *P. cinnamomi* MAT A1 and MAT A2 populations

In this study, both MAT A1 and A2 were present in Australia, Indonesia, Japan, Papuan New Guinea, South Africa, Taiwan, and Vietnam, whereas MAT A1 was absent in North and South America and Europe. A2 isolates show higher viral diversity and abundance across diverse global regions (495 infections, ∼76.4% of all infections; 144 isolates; 3.44 infections per isolate) than MAT A1 (149 infections, ∼22.5%; 71 isolates; 2.10 infections per isolate) and sterile isolates (6 infections, ∼0.93%; 3 isolates; 2.0 infections per isolate) and one unknown type (SU1119) with 1 infection (∼0.15%) (Fig. 7b, c). In Southeast Asian countries (Vietnam and Indonesia), where the A1/A2 mating type ratio is nearly balanced (27:34) (Table S1), the prevalence of viruses in both mating types is comparable (75:85 total virus infections = 2.7/2.5 infections per isolate) albeit A2 isolates exhibit a slightly broader spectrum of virus types (Fig. 7b, S1). Notably, Indonesian A2 populations distinguish themselves as hosts for the highest diversity of viruses within the region, followed by Indonesian A1, Vietnamese A1 and A2, and lastly, Taiwanese A1 and A2 populations. Japanese (pools 20 and 21) and Papuan (pool 04) A1 and A2 isolates also host viruses, whereas Australian and South African A1 isolates are virus-free. Conversely, outside of Asia and Papua New Guinea (Oceania) 100% of the MAT A2 isolates host double or multiple viral infections (Figure S1).

### Intraspecific genetic variability of locally and globally distributed viruses

The distribution patterns of various virus species among *P. cinnamomi* A2 isolates unveil interesting insights into their global spread. Notably, bunyavirids such as PciBLV3, PciBLV4, narnavirids-like PciNLV2, and the ormycovirids PciOMV1, PciOMV2, PciOMV3, PciOMV7, and PciOMV9 show international distribution and are predominantly carried by A2 isolates. Among these, PciOMV1 emerges as the most prevalent and abundant, followed closely by PciOMV2. However, a notable exception exists in Vietnam, where PciBLV3 and PciBLV4 are absent, contrasting with their presence in Taiwan and Indonesia. This geographical disparity underscores the complex dynamics of viral dissemination and highlights distinct regional variations in viral populations and their associated host isolates.

**Figure 8.**
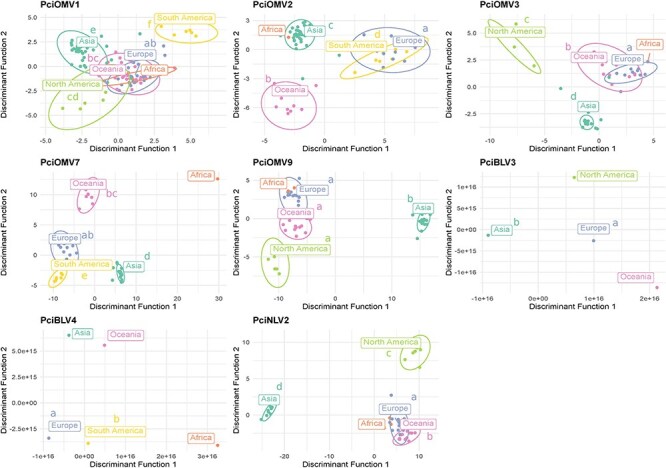
**Continent-wise DAPC + AMOVA analyses of *P. cinnamomi* viruses**. Two discriminant functions with the highest eigenvalues are displayed. Continents not sharing any lower-case letter significantly differ (AMOVA, *P* < .05). Countries for which <5 samples were available were excluded from the significance test.

Relationships between populations of the same virus across different continents can be inferred from the DAPC scatter plot shown in Fig. 8. Although the genetic differences between populations from different continents vary for each of the studied viruses, some general patterns exist. The isolates from Africa, Oceania, and Europe tend to create one cluster whereas Asian isolates always constitute a clearly defined separate cluster significantly different from all other continents (AMOVA + permutation test: *P* < .01 for the vast majority of comparisons) (Table S12). In the case of PciOMV1, the separation of Asia is less pronounced; nonetheless, despite being close to both the Africa–Europe–Oceania and the North America clusters, it is visually and statistically significantly distinct (AMOVA + permutation test: *P* < .01 for all comparisons) (Fig. 8). The Oceanian population is always significantly different from the South American one (AMOVA + permutation test: *P* < .01 for all the comparisons). The North American population is significantly different from the South American one—with PciOMV1 being the only virus shared by both populations (AMOVA + permutation test: *P* < .01).

The differentiation between populations from different countries based on the DAPC analysis is shown in Figure S8. In general, populations from only a few countries show markedly high genetic distance from the rest, namely Taiwan, Papua New Guinea, Japan, and Vietnam. Other differences are comparatively less pronounced, but the vast majority of comparisons (with at least five samples being shared by the countries compared) are significant according to the AMOVA analysis with permutation test. The results of this analysis are virus-dependent, but the following general patterns are observed. For PciOMV2 and PciNLV2, all comparisons were significant (Table S13). Two country pairs show non-significant differences for three viruses—Australia–Italy for PciOMV1, PciOMV3, and PciOMV9; and Indonesia–Portugal for PciOMV1, PciOMV3, and PciOMV7. PciOMV3 shows the highest cross-country genetic stability, with 8 out of 28 comparisons being non-significant. The PciBLV3 and PciBLV4 viruses were not included in the AMOVA analysis due to insufficient numbers of isolates—every comparison included a country that contained <5 samples.

Additionally, average pairwise identity of the alignments of the partial viral sequences (variants) obtained by RT-PCR for all viruses is shown Table S14.

## Discussion

### Genomic diversity and phylogenetic insights into oomycete viruses

The majority of the viruses identified in *P. cinnamomi* share significant genomic features with viruses previously observed in other *Phytophthora* species and oomycete genera. Thus, (+)ssRNA viruses, namely endornaviruses and narna-like viruses, are commonly detected infecting oomycetes (Poimala et al. 2021; Uchida et al. 2021; Botella et al. 2022; Raco et al. 2022, 2023; Forgia et al. 2024). Although less common, ambiguiviruses and tombus-like viruses have also been documented in these organisms (Yokoi et al. 2003; Grasse and Spring 2015; Chiapello et al. 2020; Botella et al. 2022). Furthermore, dsRNA viruses, such as fusagraviruses and toti-like viruses, showcase considerable diversity within oomycete populations (Cai and Hillman 2013; Sasai et al. 2018; Chiapello et al. 2020; Botella and Jung 2021; Poimala et al. 2021; Botella et al. 2022; Raco et al. 2022; Xu et al. 2022; Forgia et al. 2024). Notably, our study unveils the first finding of an alphachrysovirus in a *Phytophthora* species, thereby broadening our understanding of dsRNA virus diversity within this group. Oomycete viruses often exhibit shared genomic characteristics with viruses infecting fungi, invertebrates, and plants, as supported by phylogenetic evidence. However, they also display distinct phylogenetic lineages (see Figs 1–3, 6, S3), as exemplified by alphaendornaviruses, narnaviruses, fusagraviruses, and toti-like viruses, the latter recently partially classified into the family *Ootiviridae* (Sato et al. 2023). For instance, PciTLV1 clearly clusters with ootivirids, although its genome size exceeds the typical size range observed for this group (up to 5.9 kb) (Sato et al. 2023). Conversely, PciTLV2 possesses a smaller genome (5.5 kb) but falls within a diverse cluster alongside unclassified toti-like viruses infecting plants, fungi, algae, and other oomycetes.

Of particular interest are bunya-like viruses, which are highly prevalent across various oomycete species, as demonstrated by studies on the ‘*P. palustris*’ complex (Botella et al. 2022), *P. condilina* (Botella and Jung 2021), *P. castaneae* (Raco et al. 2022), *P. cactorum* (Poimala et al. 2021), *Halophytophthora* spp. (Botella et al. 2020), and *Plasmopara viticola* (Chiapello et al. 2020). Their abundance and diversity raise intriguing questions about their origins and classification as they frequently cluster into distinct evolutionary lineages. Notably, although bi- and tripartite bunya-like viruses have been described in cogu-like viruses identified in *Pl. viticola*-associated lesions from grapevines, infections of pure oomycete mycelial cultures often appear to lack second segments, with only one instance of a nucleocapsid protein (NC) observed in a putative phenuivirus in a pure culture of the ‘*P. palustris*’ complex (Botella et al. 2022). This suggests that these missing segments may either evade detection due to limitations in homology-based identification methods using the GenBank databases or may be absent. The absence of segments encoding NCs could potentially be attributed to the unique configuration of the RdRP, which may not require the conventional ribonucleoprotein particles’ formation for successful replication (Pagnoni et al. 2023). Accordingly, certain fungal bunya-like viruses found in *Tulasnella* spp. have been reported to harbor tricistronic genomes, featuring ORFs for both putative RdRP and putative NC in an ambisense orientation on the same genomic RNA, as elucidated by Sutela et al. (2020). Although our study, employing diverse bioinformatics’ pipelines, successfully identified a diverse array of novel bunya_RdRPs (18 putative species), it was unable to detect or identify potential NCs. If certain bunya-like viruses in oomycetes indeed lack a capsid a potential scenario is suggested where bunya-like viruses infecting other eukaryotes may have evolved to acquire capsids from their hosts (Krupovic and Koonin 2017; Koonin et al. 2022). Future efforts should focus on demonstrating that the L segment alone can function as a fully competent RdRP using an infectious cDNA clone, providing the first evidence that certain *Bunyaviricetes* members can replicate without the nucleocapsid.

### One potential cross-kingdom plant-oomycete infection

One panicovirus (family *Tombusviridae*) was identified in two Chilean *P. cinnamomi* isolates. PciTbLV1 appears to be 99% identical to BGLV, a virus infecting Bermuda grass (*Cynodon dactylon* L.) in the USA (Tahir et al. 2017) and Australia (Tran et al. 2022). BGLV virions have been observed in both symptomatic (chlorosis) and asymptomatic plants, but a correlation between the presence of the virus and symptoms could not be established, and the infection was, therefore, considered to be latent. The similarity between PciBLV1 and BGLV raises the possibility of a potential cross-kingdom infection with unclear origin. The fact that all Chilean *P. cinnamomi* isolates included in this study belonged to the globally distributed clonal lineage PcG2-A2 (Shakya et al. 2021) suggests that the introduced ancestor of these *P. cinnamomi* isolates might have already been infected by the virus in its area of origin. *P. cinnamomi* primarily infects woody plants, particularly those with susceptible root systems, but it was also demonstrated causing both non-symptomatic and symptomatic infections of roots in a wide range of annual and herbaceous perennial plants in Western Australia (Crone et al. 2013a, 2013b) and of herbaceous species within the genus *Lupinus* (Serrano et al. 2010). Therefore, it seems possible that the Bermuda grasses infected by BLGV in the USA and Australia were also infected by *P. cinnamomi*, and this potential infection may have gone undetected due to its symptomless nature. Alternatively, it cannot be ruled out that the potential cross-kingdom transmission happened after the introduction of *P. cinnamomi* to Chile. Bermuda grass (*C. dactylon*) is native to Europe, Africa, Australia, and much of Asia and has been introduced to the Americas. Together with *C. dactylon* × *Cynodon transvaalensis* hybrids, it is among the most widely planted turf grass species in warm-temperate, tropical and subtropical areas for golf greens, sport fields, and parks (Haydu et al. 2008). Despite its location in a temperate climate, the Valdivian rainforest is considered a tropical rainforest in a non-tropical climate originating as a Tertiary relic from the supercontinent Gondwana (Seibert 1996). It is conceivable that in the Valdivian rainforest root systems of grass species related to Bermuda grass or other herbaceous species could serve as hosts for both PciTbLV1 and *P. cinnamomi* or that PciTbLV1 might have been present in roots of the trees infected with the collected isolates of *P. cinnamomi: Castanea sativa, Drimys winteri, Luma apiculata*, *Saxegothaea conspicua*, and *Nothofagus dombeyi* (Table S1). Further research is needed to elucidate this potential cross-kingdom transmission.

Although virus transmission between plant and oomycete hosts has not been directly demonstrated in nature, some viruses and their hosts have been experimentally shown to cross the barrier between kingdoms under suitable conditions, such as close association/contact and compatibility with a potential new host (Liu et al. 2016; Mascia et al. 2019; Bian et al. 2020; Telengech et al. 2024). Some fungal viruses infecting marine fungi associated with seagrass have been shown to replicate in plant protoplasts (Nerva et al. 2017). Evidence of virus transmission from plant to fungus is provided by the natural infection of the fungus *Rhizoctonia solani* with the plant virus cucumber mosaic virus (Andika et al. 2017). Moreover, artificial virus inoculation experiments have demonstrated the compatibility of fungi as hosts of diverse plant viruses (Andika et al. 2023). Viruses crossing kingdoms are expected to be crucial players in ecosystem balance (Kondo et al. 2022; Telengech et al. 2024) and it is suggested that RNA viruses have likely undergone complex evolutionary pathways, potentially involving host switches and adaptation to new environments (Sanjuán and Domingo-Calap 2016). Accumulating evidence of cross-kingdom virus infections with different types of RNA viruses offers a perspective on the evolutionary history of RNA viruses and their interactions with diverse host organisms

### Highly diverse and prevalent ormycovirid infections

*Phytophthora cinnamomi* populations exhibit an exceptional abundance and diversity of ormycoviruses, comprising 12 putative species. Ormycoviruses were initially documented in the yeast *Starmerella bacillaris* (Crucitti et al. 2022) and in phytopathogenic fungi, oomycetes and ectomycorrhizal fungi by Forgia et al. in 2022. Subsequent research has expanded their known distribution, with reports of ormycoviruses in several fungal species, including *Trichoderma tomentosum* (Pagnoni et al. 2023), *Verticillium dahliae* (unpublished, GenBank accession WPV08068) and the ectomycorrhizal fungus *Hortiboletus rubellus* (Sahin et al. 2024), and the seaweeds *Alaria esculenta* and *Saccharina latissima* (Dekker et al. 2024, preprint). Interestingly, ormycoviruses are notable for lacking the canonical ‘GDD’ catalytic triad in their putative RdRps (Olendraite et al. 2023). Instead, prevalent putative catalytic triads observed include ‘NDD’ (more predominant in Alpha- and Beta-OMVs) and ‘GDQ’ (typically found in Gamma-OMVs), with lesser occurrences of ‘SDD’, ‘HDD’, and ‘ADD’ (G, Glycine; D, Aspartic acid; Q, Glutamine; N, Asparagine; S, Serine; H, Histidine; A, Alanine). PciOMVs belong to the *Alpha*- and *Deltaormycoviridae* families, predominantly exhibiting ‘NDD’, ‘SDD’ is observed in two of them (PciOMV9 and 10), which are member of the genus *Dormycovirus*. Further investigation into these novel taxa revealed supporting evidence for the presence of a second RNA segment encoding a conserved protein. However, it is still impossible to correctly assign a function to the HPs coded by PciOMVs as the structural models reveal poor conservation among them and no significant results could be obtained when submitting the models on the DALI and Foldseek servers (data not shown). RNA2 was found in all PciOMVs but two (PciOMV8 and 10), due to the diversity of orphan contigs in the correspondingly containing pools (P4, 13, 14, and 15); however, we cannot rule out their presence in our data. PciMOMV1, which exhibits both ORFs within the same segment, suggests it could have originated from a likely recent recombination event, given its shared RdRp-ORF1 and HP-ORF2 with PciOMV6. Alternatively, PciMOMV1 might represent an intermediate state during replication, packaging, or defective genome formation. Although a rare event, some RNA viruses, including reoviruses, may organize their genomes into a single linear genome through duplication and recombination, allowing the virus to encode all necessary functions in one segment (Smith et al. 2021). In other cases, such as tombusviruses, the recombination between defective RNAs generates functional hybrid genomes (White and Morris 1994). Viral replication often involves the synthesis of complementary RNA, and in segmented viruses, these intermediate molecules might temporarily appear as separate fragments. Since no ribozymes were detected, a viroid-like RNA nature can likely be excluded. This raises the possibility that PciMOMV1 might represent an intermediate stage of PciOMV6. Nevertheless, we cannot exclude the possibility that the amplification is merely an artefact resulting from potential 5′ and 3′ end complementarity, which could facilitate the overlapping of RNA1 and RNA2 at the 5′–3′ end junction. However, further empirical evidence is needed to confirm this hypothesis.

### *Phytophthora cinnamomi*’s viral epicentre is in Southeast Asia

The origin of *P. cinnamomi* has sparked considerable debate over many years, with increasing evidence pointing to its likely emergence in Southeast Asia or Taiwan (Pratt et al. 1973; Ko et al. 1978; Arentz and Simpson 1986; Arentz 2017; Jung et al. 2017, 2020). A recent population genomic study of a global collection of 204 *P. cinnamomi* isolates, of which 171 were also included in the present study, found the highest genotypic diversity and a partially sexual semi-clonal mode of reproduction in the populations from Taiwan and Vietnam indicating these regions are within or near the centre of origin (Shakya et al. 2021). Understanding the geographic distribution of virus diversity can offer valuable insights into the origin of *P. cinnamomi*. Higher diversity of a specific virus or group of viruses in one region as compared to others suggests a potential origin or significant presence in this region (Roderick and Navajas 2003; Feau et al. 2014; Schoelz and Stewart 2018; French and Holmes 2020; Botella et al. 2022). Our findings concur with this notion, as Asia emerges as the epicentre of viral diversity within *P. cinnamomi*. Within Asia, the *P. cinnamomi* populations of Indonesia and Vietnam exhibit the highest diversity of virus types, while Taiwan stands out for its abundance of viral infections. Interestingly, this trend may reduce towards higher latitudes in both hemispheres. The Japanese population shows a decrease in both virus abundance and diversity whereas in Australia the viral abundance is very high, but the diversity of virus types and species richness aligns closely with other introduced regions worldwide, such as Japan and Portugal. Similarly, in the ‘*P. palustris*’ complex viruses were absent in Japan and Taiwan, while Indonesian populations in Kalimantan and Sumatra hosted a highly diverse virome (Botella et al. 2022). Such geographical patterns suggest that not all viruses hosted in native oomycete communities successfully establish themselves when their hosts are spread to other biogeographic regions.

The introduction of *P. cinnamomi* into new territories like Africa, Australia, Europe, North, or South America has apparently led to founder effects resulting in both a genetic bottleneck of the pathogen (Dobrowolski et al. 2003; Shakya et al. 2021) and a bottleneck in virus diversity. Interestingly, contrary to viral diversity, the number of viral infections per isolate outside Asia increased in favour of seven viruses, including bunyavirids PciBLV3 and PciBLV4, narnavirid PciNLV2, and ormycovirids PciOMV1, PciOMV2, PciOMV3, PciOMV7, and PciOMV9, which exhibit a widespread distribution, indicating a deep connection with their host *P. cinnamomi*. Most likely, this robust association might stem from long-term coevolution in their native environment. Despite numerous introductions into new regions, geographical separation, and host diversification, this connection remained resilient. Indeed, the efficient transmission of these viruses alongside their host implies either a commensalistic, an ammensalistic or a mutually beneficial interaction rather than a detrimental one. It also suggests that these viruses might have a role in shaping *P. cinnamomi* behaviour and adaptation, strategically adjusting transmission rates to ensure their continued spread and survival within host populations as it has been the case for CHV1 (Bryner et al. 2012). This hypothesis is supported by the fact that, on a global scale, these viruses are only carried by *P. cinnamomi* MAT A2 isolates, responsible for the pandemics in Australian, European, Chilean, and/or USA woodlands and crops (Kamoun et al. 2015; Hardham and Blackman 2018; Jung et al. 2018a, 2018b; Serrano et al. 2019; Shakya et al. 2021).

The presence of both MAT A1 and A2 in native stable populations, such as those in Indonesia, Taiwan, and Vietnam, appears to foster sexual recombination, as indicated by simulations based on the index of association (Shakya et al. 2021), and, hence, the opportunity for virus exchange between mating types. In these native regions, the presence of both mating types in an approximately balanced ratio likely promotes a dynamic virome, with viruses being naturally transmitted between MAT A1 and A2. Among many examples (Figure S1), the fusagra-like virus 1 (PciFLV1) is hosted by one A1 and two A2 isolates from Sumatra, and one Vietnamese A1 isolate. However, in regions of *P. cinnamomi* introduction like Australia and South Africa, despite the coexistence of both MATs, there is no evidence of successful sexual reproduction between A1 and A2 types (Dobrowolski et al. 2003; Shakya et al. 2021). The absence of viruses in MAT A1 isolates and the lack of sexual crossing outside Asia suggest the source of viruses in MAT A1 is the sympatric presence of MAT A2, enabling virus transmission via the transfer of cytoplasm and nuclei from the male antheridium to the female oospore during sexual reproduction.

### Virome evolution across local and global populations of *P. cinnamomi*

In regions where both mating types of *P. cinnamomi* mate, the resulting genetic recombination could produce also a wider variety of viral genotypes (Nagy and Simon 1997). This could explain the higher viral species richness in the native Asian range, where sexual outcrossing of the host *P. cinnamomi* is more common (Jung et al. 2017, 2020; Shakya et al. 2021). In contrast, regions with predominantly asexual reproduction and sexual inbreeding via the selfing of MAT A2, such as Australia, Africa, Chile, Europe, and the Americas, show lower viral diversity, supporting the idea that sexual outcrossing of the host facilitates viral diversification. The interaction between *P. cinnamomi* and its associated viruses suggests a dynamic coevolution. As the host undergoes genetic changes through sexual outcrossing and recombination, viruses must adapt to new host genotypes, leading to accelerated viral evolution (Sanjuán and Domingo-Calap 2016).

Accordingly, based on the DAPC analyses, the eight viruses with global distribution (PciOMV1, 2, 3, 7, and 9; PciBLV3 and 4 and PciNLV2) show stable genetic structure across continental populations and a certain degree of divergence. Navigating heterogeneous environments due to host shifts and geographical heterogeneity, RNA viruses accumulate genomic mutations, predicted to have significant consequences for their evolution (Sanjuán and Domingo-Calap 2016). Although in fungal and oomycete viruses the transmission is intracellular (Ghabrial and Suzuki 2009), there is also a certain degree of variability, as seen in the differences between continents (and countries). The DAPC analyses further suggest that these RNA viruses follow distinct evolutionary patterns, likely due to varying mutation rates, even when co-existing within the same *P. cinnamomi* isolates. The most distinctive viral populations are found in Taiwan, Vietnam, and Japan while Indonesian populations more closely resemble the panglobal clonal populations. This suggests Indonesia being the most likely geographical origin of the viral populations and, consequently, the two clonal *P. cinnamomi* lineages that have spread across the world. This is in agreement with the branch lengths of globally distributed viruses compared to native Taiwanese, Vietnamese, and Indonesian virus species in the phylogenetic trees of the distinct viral RdRPs. Longer branches in Indonesia, Taiwan, Sumatra, and Papua New Guinea suggest significant evolutionary changes over longer time periods, leading to the emergence of different species, i.e. PciOMV4 and 5, PciOMV6 and 11, and PciOMV9 and 10. In contrast, the shorter branch lengths of viral species and their variants in introduced clonal populations of *P. cinnamomi* suggest a very short evolutionary period, indicating a recent introduction of these viruses alongside their host into new territories.

## Conclusions

This study provides the first comprehensive analysis of the virome of *P. cinnamomi*, a major global plant pathogen. We identified a high prevalence of novel RNA viruses across isolates from various continents, with the highest viral diversity found in East and Southeast Asia, pointing to this region, and particularly, Indonesia, as the origin of *P. cinnamomi*. Eight globally distributed viral species show genetic divergence yet overall stability. Host sexual recombination appears to enhance viral diversity, especially in native regions where sexual outcrossing between both mating types occurs, while in introduced regions with predominantly asexual reproduction and occasional inbreeding of the A2 mating type significantly fewer virus species occur. Furthermore, the study highlights the role of the A2 mating type in spreading viruses across regions and also suggests potential cross-kingdom infections.

## Supplementary Material

veaf020_Supp

## Data Availability

Amplicon sequences data and agarose gel pictures are available at https://figshare.com/account/items/26029246/. 10.6084/m9.figshare.26029246. SRA records are accessible in the bioproject PRJNA1102859 using the link https://www.ncbi.nlm.nih.gov/sra/PRJNA1102859. Virus/contig sequences were registered in the GenBank sequence database under the accession numbers PP891625–PP891946.
