# Supplementary material for: The virome of the panglobal, wide host-range plant pathogen Phytophthora cinnamomi: phylogeography and evolutionary insights
Source: Virus Evol. 2025 Apr 1;11(1):veaf020. doi: 10.1093/ve/veaf020 (PMC12063590; doi:10.1093/ve/veaf020)
Supplement: veaf020_Supp [file veaf020_supp.zip › suppl_data/Figure S2.PciTbLV1 coverage.pdf]

(a)

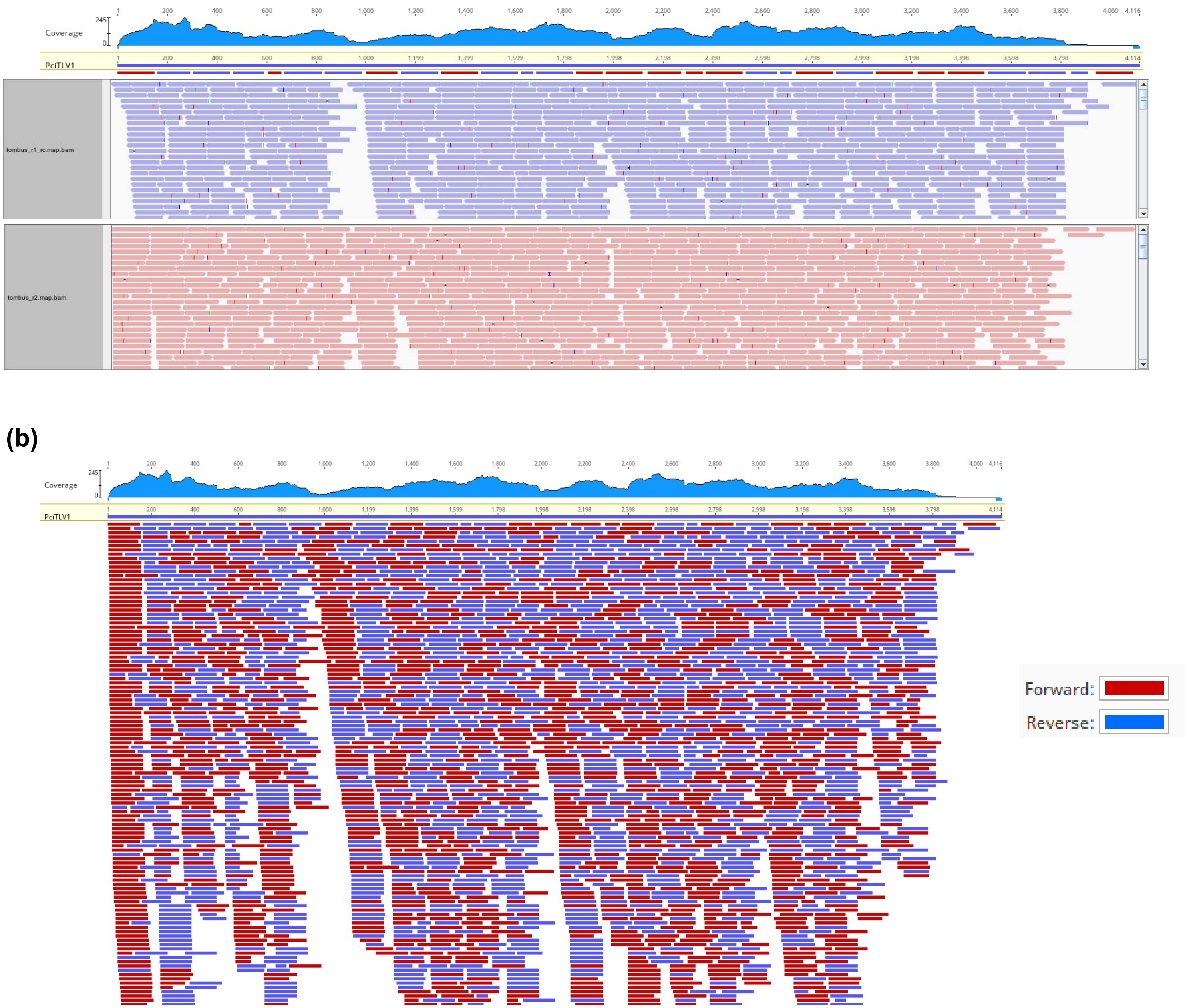

Coverage of 4,111 bases:
Mean: 108.2 Std Dev: 49.5
Minimum: 0 Maximum: 245
Forward: 54.6 % Reverse: 53.5 %

Figure S2. Coverage Plots illustrating the read sense in IGV (a) and in Geneious Prime (b) for the de novo assembly of PciTlV1**.**
