# Supplementary material for: The virome of the panglobal, wide host-range plant pathogen Phytophthora cinnamomi: phylogeography and evolutionary insights
Source: Virus Evol. 2025 Apr 1;11(1):veaf020. doi: 10.1093/ve/veaf020 (PMC12063590; doi:10.1093/ve/veaf020)
Supplement: veaf020_Supp [file veaf020_supp.zip › suppl_data/Figure S3.Cladogram PciNLV1-8.pdf]

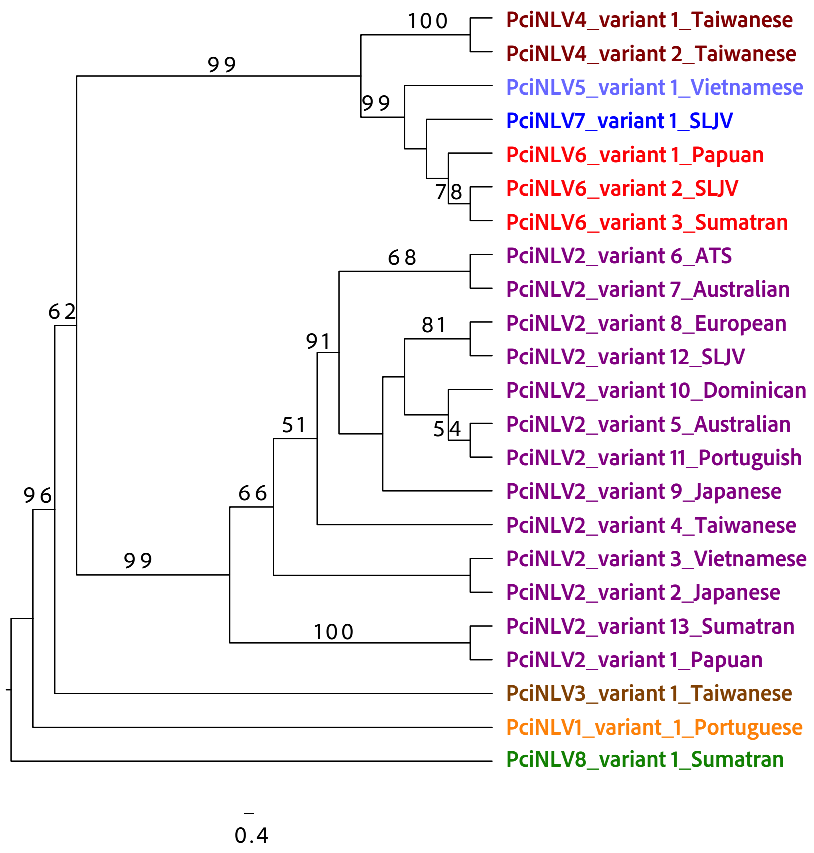

Figure S3 . RAxML cladogram illustrating the phylogenetic relationships of PciNLV1-8. Branch lengths are proportionally scaled to the anticipated number of amino acid substitutions per site. Nodes are annotated with bootstrap support values ≥50%. Branch lengths are proportionally scaled to the anticipated number of amino acid substitutions per site. Phytophthora cinnamomi narna-like viruses 1—8 (and variants) are denoted by their abbreviated names. The scale bars indicate expected changes per site per branch.
