# Supplementary material for: The virome of the panglobal, wide host-range plant pathogen Phytophthora cinnamomi: phylogeography and evolutionary insights
Source: Virus Evol. 2025 Apr 1;11(1):veaf020. doi: 10.1093/ve/veaf020 (PMC12063590; doi:10.1093/ve/veaf020)
Supplement: veaf020_Supp [file veaf020_supp.zip › suppl_data/Figure S4. -ssRNA.Bunyaviruses.pdf]

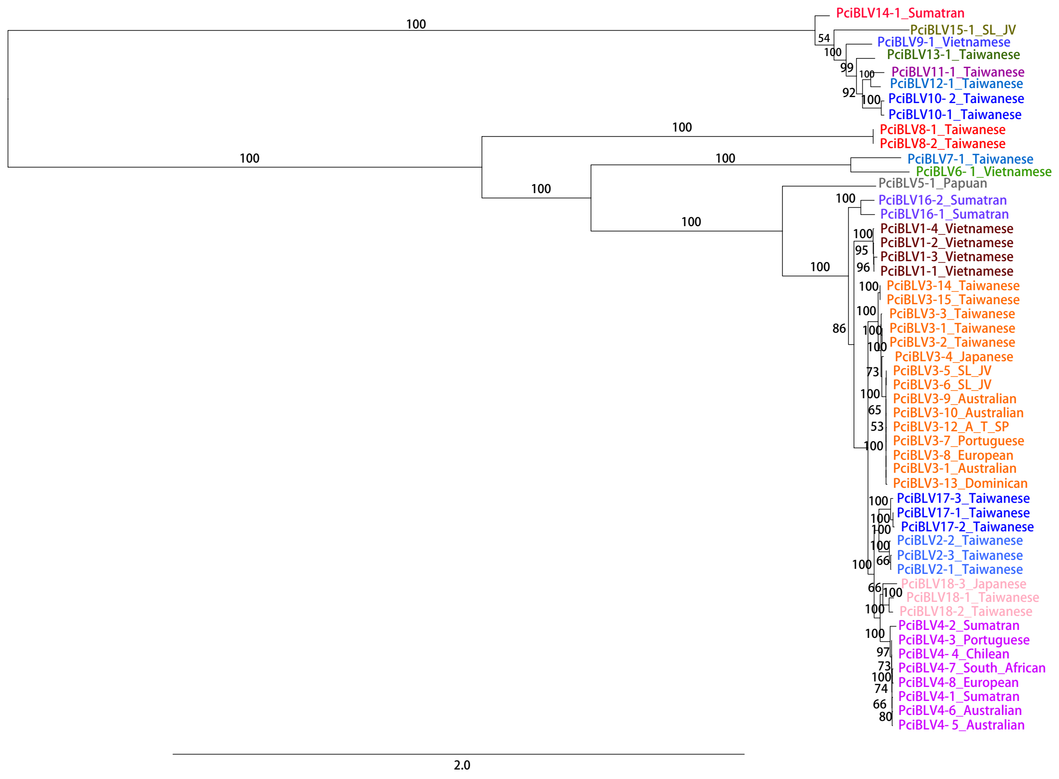

Figure S4. RAxML tree shows the phylogenetic relationships of the predicted RdRP of all the species and variants of PciBLV1-18. Viral variants are distinguished based on colors, the variant name and origin of the host are indicated. SL_JV means the variant was found in the RNA pool with Sulawesian and/or Javanese origin (P14); A_T_SP means Algerian-Tunisian and/or Spanish origin (P03). Nodes are labeled with bootstrap support values ⩾50%. Branch lengths are scaled to the expected underlying number of amino acid substitutions per site. Tree is rooted in the midpoint. Scale bar = 2.0 expected changes per site per branch.
