# Supplementary material for: The virome of the panglobal, wide host-range plant pathogen Phytophthora cinnamomi: phylogeography and evolutionary insights
Source: Virus Evol. 2025 Apr 1;11(1):veaf020. doi: 10.1093/ve/veaf020 (PMC12063590; doi:10.1093/ve/veaf020)
Supplement: veaf020_Supp [file veaf020_supp.zip › suppl_data/Figure S5. Cladogram OMV RNA 2.pdf]

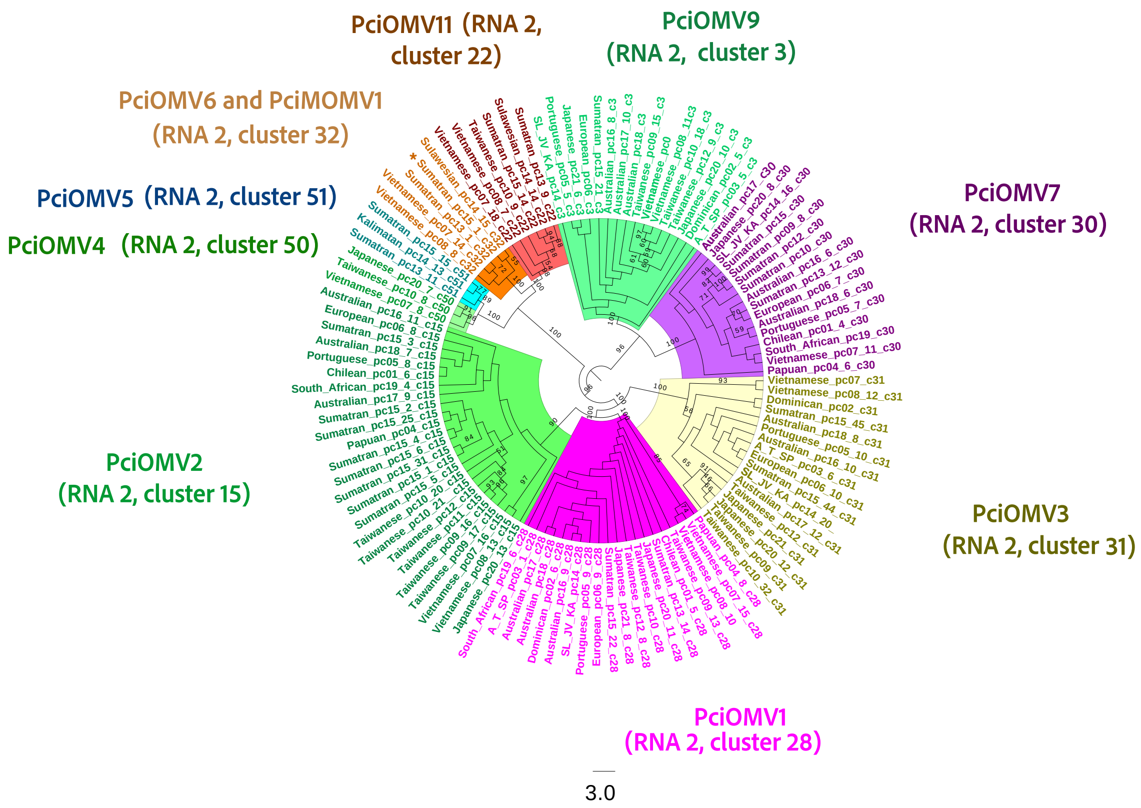

Figure S5. Circular RAxML cladogram tree based on the predicted HP of *Phytophthora cinnamomi* ormycoviruses′ contigs (PciOMV1-11). Species and variants are color-coded. Variants names include the country of origin of *P. cinnamomi* host, the RNA pool number where they were detected (pc) and the cluster of nucleotide and protein similarity % initially assigned, necessary for the species description. More information can be found in Table 1 and Table S3.
