# Supplementary material for: The virome of the panglobal, wide host-range plant pathogen Phytophthora cinnamomi: phylogeography and evolutionary insights
Source: Virus Evol. 2025 Apr 1;11(1):veaf020. doi: 10.1093/ve/veaf020 (PMC12063590; doi:10.1093/ve/veaf020)
Supplement: veaf020_Supp [file veaf020_supp.zip › suppl_data/Figure S6. 3D Ormyc model.pdf]

(a)

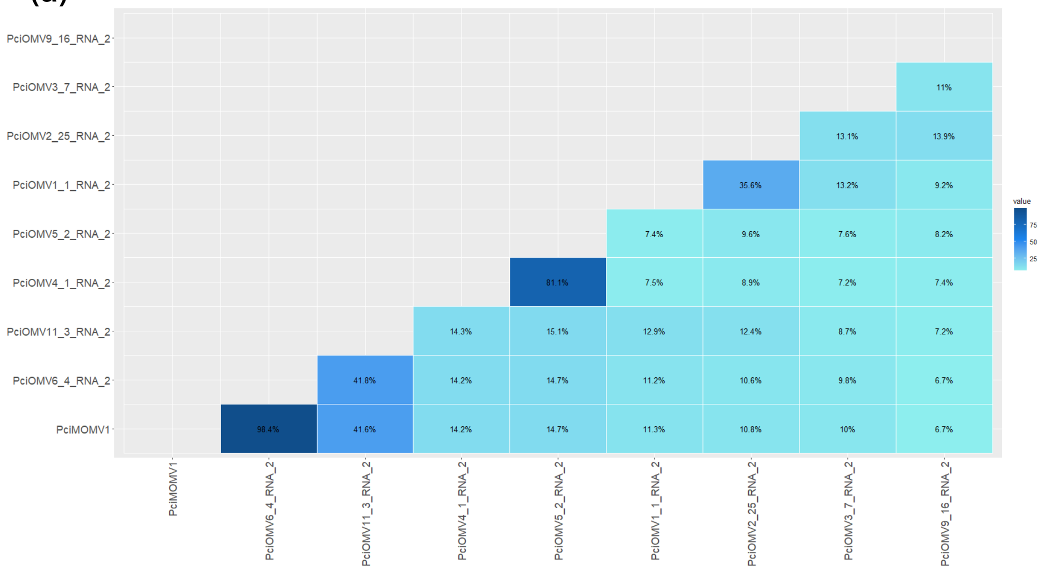

(b)

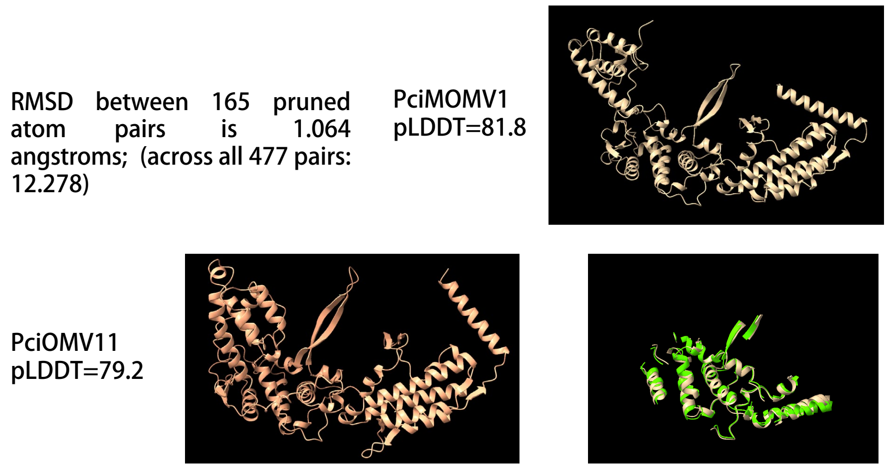

(c)

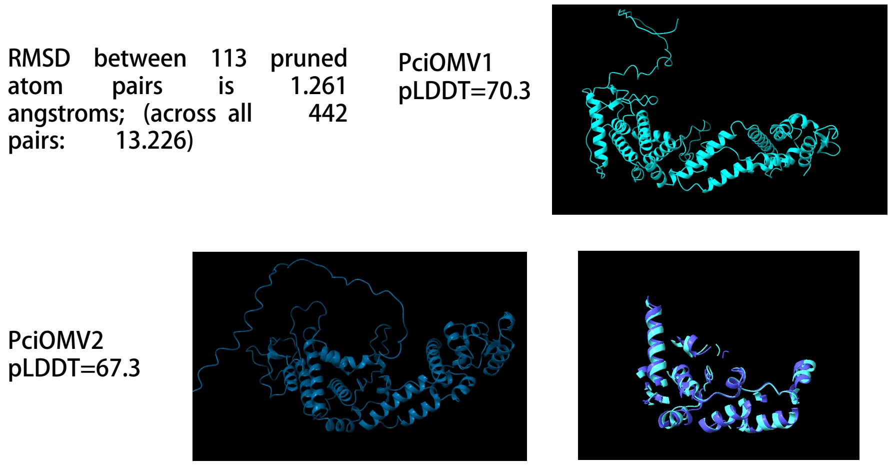

(d)

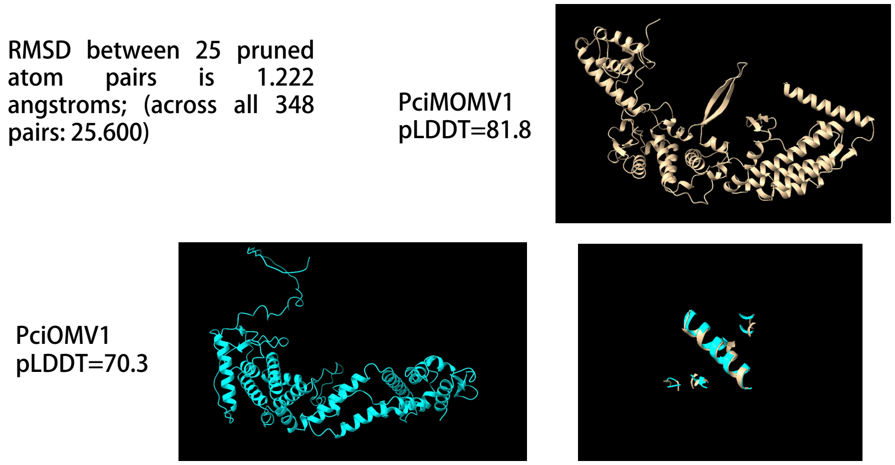

Figure S6. (a) Identity percentages and (b) (c) (d) Graphic representation of the 3D structures of selected ormycovirus RdRP and HP.
