# Supplementary material for: The virome of the panglobal, wide host-range plant pathogen Phytophthora cinnamomi: phylogeography and evolutionary insights
Source: Virus Evol. 2025 Apr 1;11(1):veaf020. doi: 10.1093/ve/veaf020 (PMC12063590; doi:10.1093/ve/veaf020)
Supplement: veaf020_Supp [file veaf020_supp.zip › suppl_data/Figure S7.PciMOMV coverage.pdf]

(a)

(b)

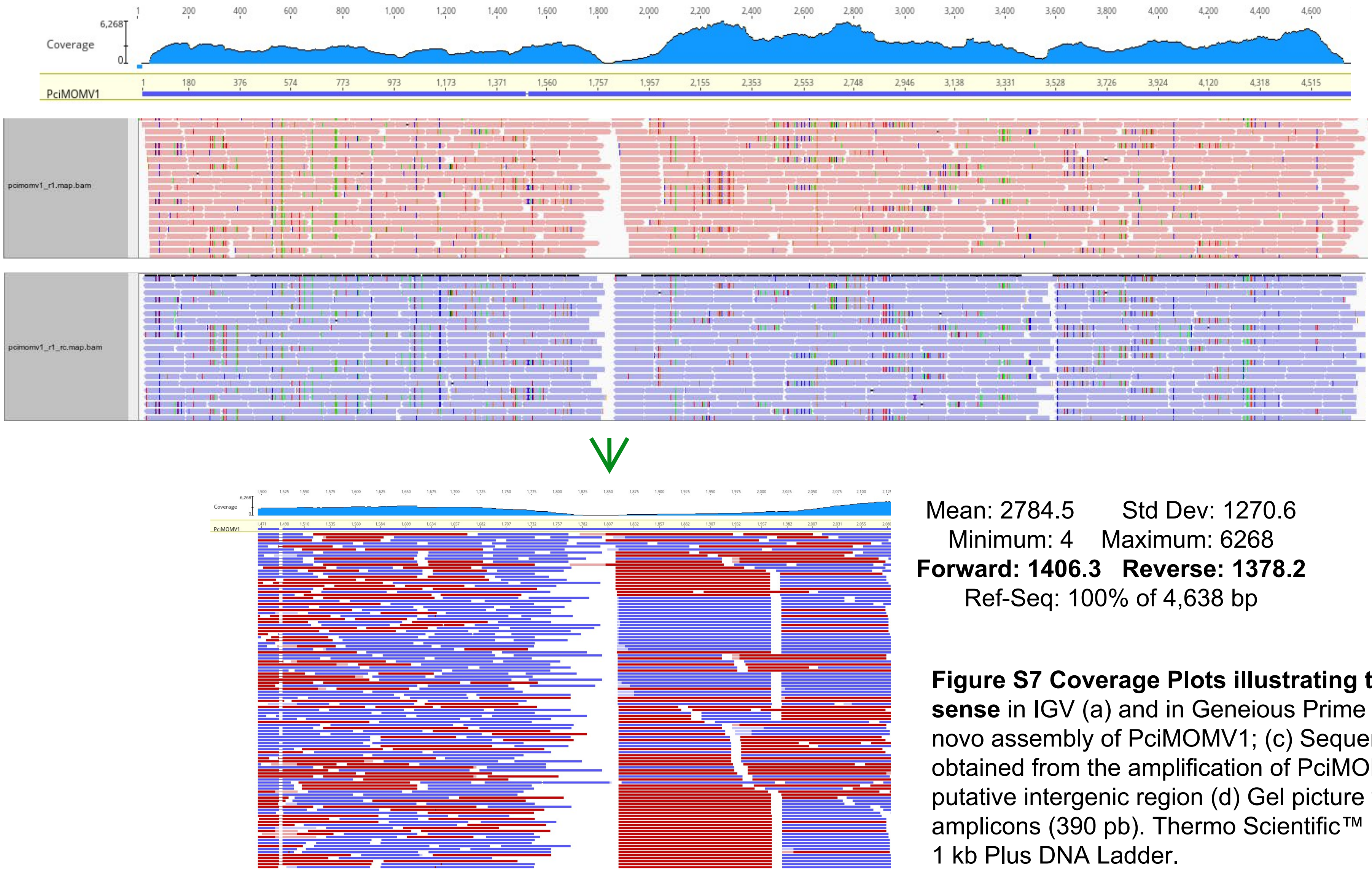

Mean: 2784.5 Std Dev: 1270.6
Minimum: 4 Maximum: 6268
Forward: 1406.3 Reverse: 1378.2
Ref-Seq: 100% of 4,638 bp

(c)

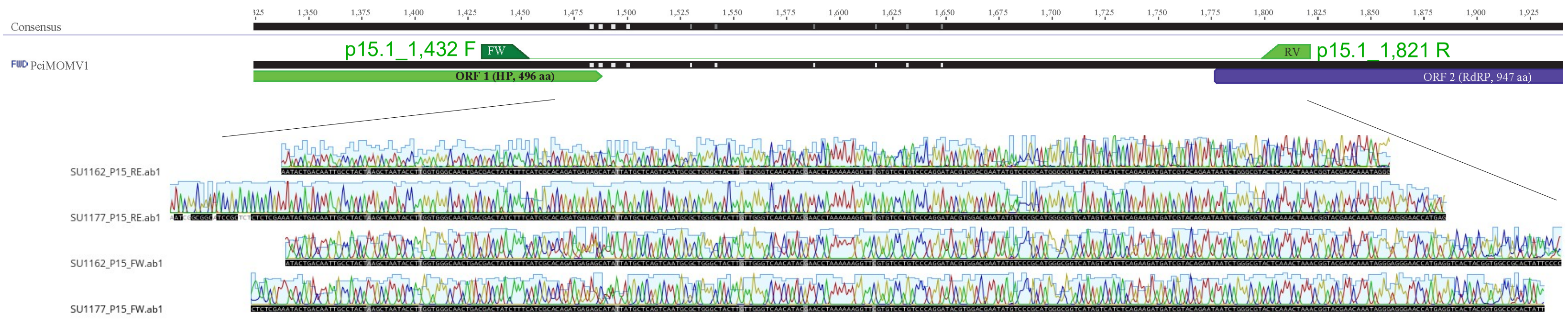

(d)

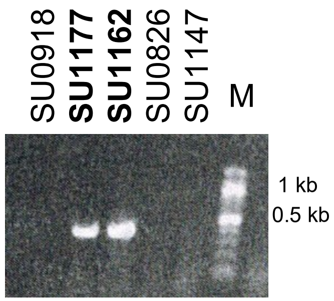

Figure S7 Coverage Plots illustrating the read sense in IGV (a) and in Geneious Prime (b) for the novo assembly of PciMOMV1; (c) Sequences obtained from the amplification of PciMOMV1 putative intergenic region (d) Gel picture with the amplicons (390 pb). Thermo Scientific™ GeneRuler 1 kb Plus DNA Ladder.
