# Supplementary material for: The virome of the panglobal, wide host-range plant pathogen Phytophthora cinnamomi: phylogeography and evolutionary insights
Source: Virus Evol. 2025 Apr 1;11(1):veaf020. doi: 10.1093/ve/veaf020 (PMC12063590; doi:10.1093/ve/veaf020)
Supplement: veaf020_Supp [file veaf020_supp.zip › suppl_data/Figure S8.DAPC_countries_new.pdf]

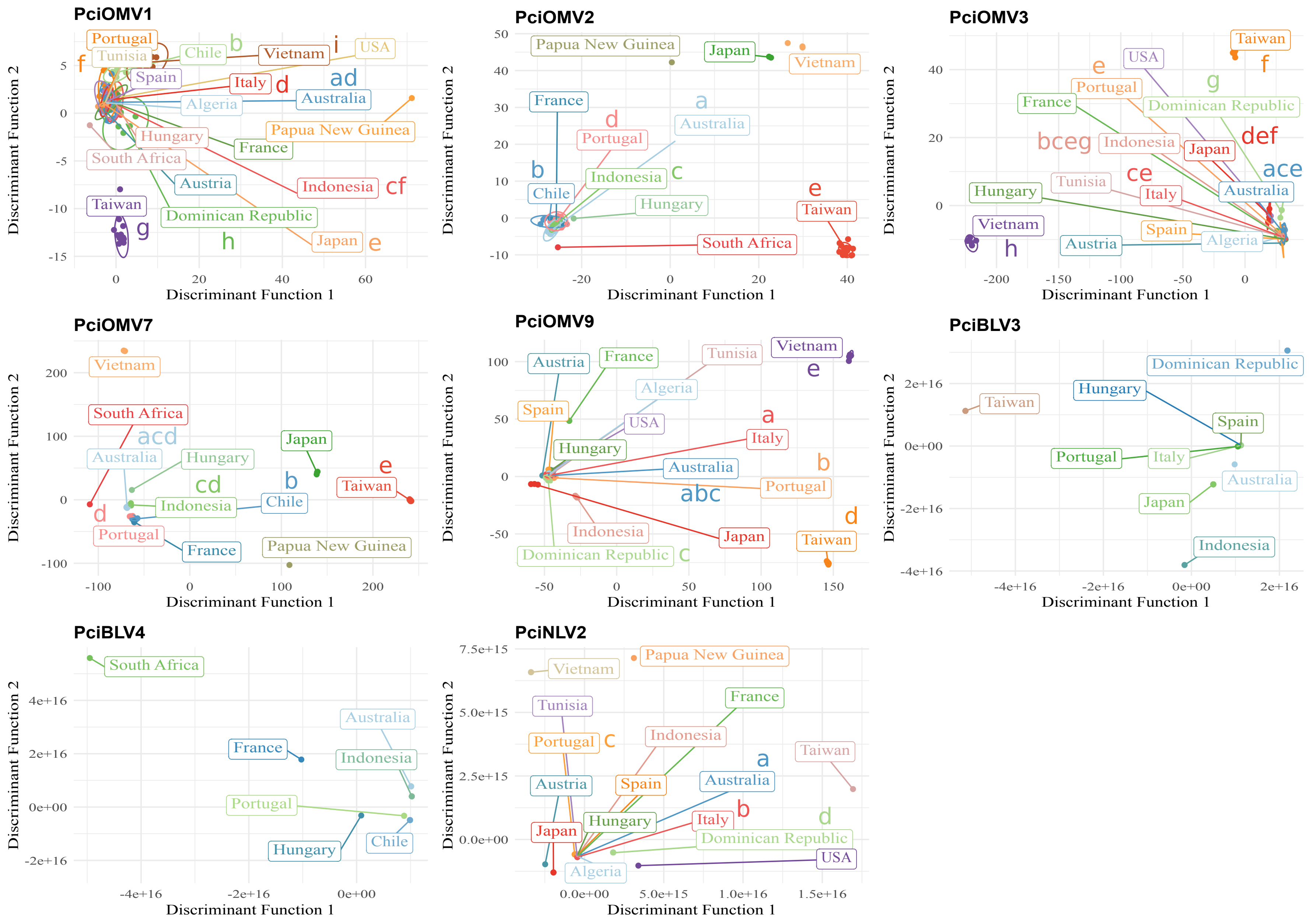

Figure S8. Country-wise DAPC + AMOVA analyses of the *P. cinnamomi* viruses. Two discriminant functions with the highest eigenvalues are displayed. Countries not sharing any lower-case letter significantly differ (AMOVA, $p < 0.05$). Countries for which less than five samples were available were excluded from the significance test.
