# Supplementary figures and images for: The virome of the panglobal, wide host-range plant pathogen Phytophthora cinnamomi: phylogeography and evolutionary insights
Source: Virus Evol. 2025 Apr 1;11(1):veaf020. doi: 10.1093/ve/veaf020 (PMC12063590; doi:10.1093/ve/veaf020)

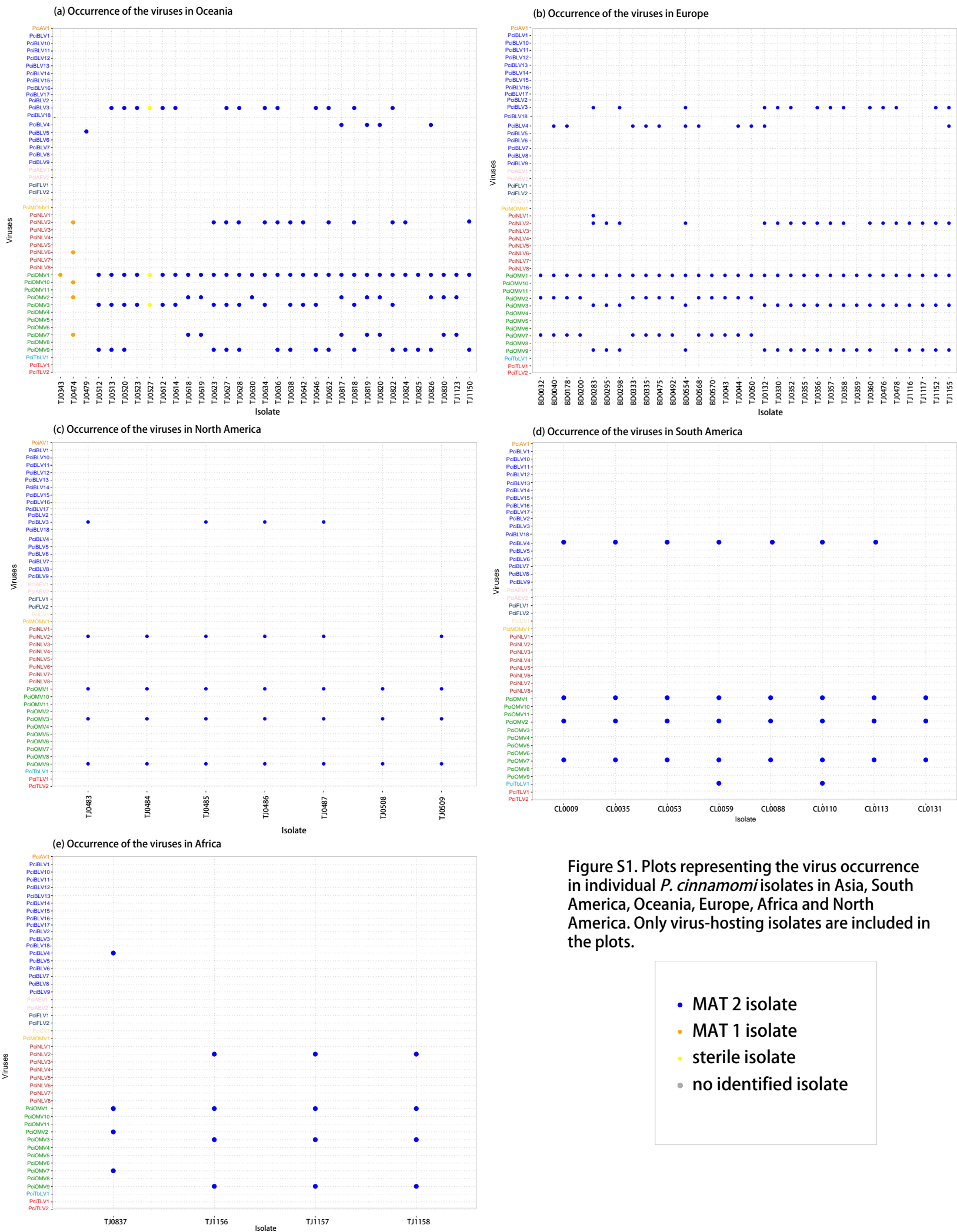

Supplement: veaf020_Supp [file veaf020_supp.zip › suppl_data/Figure S1.Occurrence OC, EU, AF, NA, SA, AS.pdf]
